# Genomic capacities for Reactive Oxygen Species metabolism across marine phytoplankton

**DOI:** 10.1371/journal.pone.0284580

**Published:** 2023-04-25

**Authors:** Naaman M. Omar, Katherine Fleury, Brian Beardsall, Ondřej Prášil, Douglas A. Campbell

**Affiliations:** 1 Department of Biology, Mount Allison University, Sackville, NB, Canada; 2 Faculty of Veterinary Medicine, University of Calgary, Calgary, AB, Canada; 3 Faculty of Computer Science, Dalhousie University, Halifax, NS, Canada; 4 Institute of Microbiology, Center Algatech, Laboratory of Photosynthesis, Trebon, CZ, Czech Republic; University of Innsbruck, AUSTRIA

## Abstract

Marine phytoplankton produce and scavenge Reactive Oxygen Species, to support cellular processes, while limiting damaging reactions. Some prokaryotic picophytoplankton have, however, lost all genes encoding scavenging of hydrogen peroxide. Such losses of metabolic function can only apply to Reactive Oxygen Species which potentially traverse the cell membrane outwards, before provoking damaging intracellular reactions. We hypothesized that cell radius influences which elements of Reactive Oxygen Species metabolism are partially or fully dispensable from a cell. We therefore investigated genomes and transcriptomes from diverse marine eukaryotic phytoplankton, ranging from 0.4 to 44 μm radius, to analyze the genomic allocations encoding enzymes metabolizing Reactive Oxygen Species. Superoxide has high reactivity, short lifetimes and limited membrane permeability. Genes encoding superoxide scavenging are ubiquitous across phytoplankton, but the fractional gene allocation decreased with increasing cell radius, consistent with a nearly fixed set of core genes for scavenging superoxide pools. Hydrogen peroxide has lower reactivity, longer intracellular and extracellular lifetimes and readily crosses cell membranes. Genomic allocations to both hydrogen peroxide production and scavenging decrease with increasing cell radius. Nitric Oxide has low reactivity, long intracellular and extracellular lifetimes and readily crosses cell membranes. Neither Nitric Oxide production nor scavenging genomic allocations changed with increasing cell radius. Many taxa, however, lack the genomic capacity for nitric oxide production or scavenging. The probability of presence of capacity to produce nitric oxide decreases with increasing cell size, and is influenced by flagella and colony formation. In contrast, the probability of presence of capacity to scavenge nitric oxide increases with increasing cell size, and is again influenced by flagella and colony formation.

## Introduction

Phytoplankton cells span a large size range, from picoplankton (<2μm), nanoplankton (2 to 20μm), microplankton (20 to 200μm) to macroplankton (200 to <2000μm) [1]. Cell size interacts with multiple selective pressures, including cellular metabolic rate, light absorption, nutrient uptake, cell nutrient quotas, trophic interactions and diffusional exchanges with the environment [1–5]. Beyond simple size, cell shapes and growth forms influence diffusional exchanges between cells and their environment [6].

### Characteristics of reactive oxygen species

Phytoplankton both produce and scavenge Reactive Oxygen Species (ROS), both within and outside the cell membrane, enzymatically and non-enzymatically. Some ROS readily cross the cell membrane, connecting intra- and extra-cellular pools. Other ROS rarely cross cell membranes and therefore intra- and extra-cellular pools are at least partially segregated (Table 1).

**Table 1:** Diffusion and stability of different ROS.

ROS	ROS Symbol	Concentration in Seawater (M)	Concentration Citation	Diffusion Distance (nm)	Diffusion Distance Citation	Lifetime (s)	Lifetime Citation	Crosses Cell Membrane	Crosses Cell Membrane Citation	Abiotic Production (Ms^-1^)	Abiotic Production Rates Citation	Diffusion Coefficient (um^2^us)	Diffusion Coefficient Citation
Hydrogen Peroxide	H_2_O_2_	10^−9^ to 10^−6^	[7, 8]	NA	NA	hours to days	[7–9]	Yes	[10–13]	1e-13	[14]	1500	[15]
Superoxide	O_2_^•−^	10^−12^ to 10^−9^	[7, 16]	320	[9]	ms to minutes	[7, 16]	No	[17]	1e-14	[14]	210	[18]
Nitric Oxide	NO^•^	10^−12^ to 10^−10^	[16, 19]	NA	NA	seconds	[16, 20]	Yes	[21]	1e-13	[16]	2210	[22]
Hydroxyl Radical	HO^•^	10^−18^ to 10^−15^	[7]	4.5	[9]	μs	[7]	No	[23]	3e-22	[24]	NA	NA
Singlet Oxygen	^1^O_2_	10^−14^ to 10^−13^	[25]	82	[9]	μs	[9]	NA	NA	NA	NA	2100	[26]
Peroxynitrite	ONOO^−^	10^−12^ to 10^−11^	[16]	NA	NA	ms	[16, 27]	Yes	[28]	1e-11	[16]	NA	NA

Superoxide (O_2_^•−^), a radical anion generated through the monovalent reduction of O_2_ to O_2_^•−^ [29], is highly reactive [30] with organic compounds including thiols [31], and with metals [32, 33]. As the first ROS in a sequential series of reductions of O_2_, O_2_^•−^ is a ‘gateway’ to production of other ROS. O_2_^•−^ is produced both inside and outside a cell [34–39], but shows limited diffusion across the hydrophobic cell membrane [17]. Multiple oxidases (S2 Table) reduce O_2_ and generate either H_2_O [40], or alternately O_2_^•−^ and/or Hydrogen Peroxide (H_2_O_2_) [41, 42]. Biogenic production of extracellular ROS is significant in marine environments [7, 43–51], and O_2_^•−^ in coastal waters is primarily attributable to extracellular production mediated by eukaryotic phytoplankton [52]. Some extracellular O_2_^•−^ production likely contributes to cell growth [53].

Two known enzymes mediate conversion of O_2_^•−^ to H_2_O_2_; the ubiquitous dismutation of O_2_^•−^ catalyzed by diverse Superoxide Dismutases (SOD) or the less prevalent reduction of O_2_^•−^, catalyzed by Superoxide Reductase (SOR) at the expense of metabolic reductant. O_2_^•−^ also dismutates spontaneously to produce H_2_O_2_ and O_2_ [54], although [55] found that ~52% of dark O_2_^•−^ production likely undergoes oxidation back to O_2_. Extracellular production of O_2_^•−^ thus contributes to extracellular H_2_O_2_ pools [47, 48, 56, 57].

H_2_O_2_ passively traverses cell membranes [58], primarily through aquaporins [10–13], allowing exchange of intracellular and extracellular pools of H_2_O_2_, although cells can maintain a concentration gradient between internal and external H_2_O_2_ [59]. H_2_O_2_ is acutely toxic to most cells in the range of 10^−5^ to 10^−4^ mol L^-1^ [58], reacting with thiols and methionine [31] and interfering with gene expression [60]. Cytotoxic effects of H_2_O_2_, including lipid damage, are however, primarily caused by H_2_O_2_ dismutating into the Hydroxyl Radical (HO^•^), which is strongly oxidative [9].

Multiple oxidases are important in producing H_2_O_2_ (S2 Table), but abiotic processes, including rainfall, may be dominant sources of extracellular H_2_O_2_ in seawater [61–63]. H_2_O_2_ concentrations in seawater follow a diurnal cycle with a peak at mid-day [61, 64, 65], suggesting significant direct or indirect photochemical or photobiological generation of H_2_O_2_. Heterotrophs do not contribute much H_2_O_2_ production but mediate H_2_O_2_ decomposition [66]. H_2_O_2_ also decomposes spontaneously, though slowly, into water and oxygen [67], and contributes significantly to the redox cycling of copper and iron in seawater [68, 69].

Despite its radical nature and ability to react with biomolecules, ^•^NO functions widely as a signaling molecule [70–72]. ^•^NO is produced both biogenically through arginine dependent Nitric Oxide Synthases (NOS) or Nitric Oxide Associated Proteins (NOA) [73], as well as through abiotic processes including nitrite photolysis [74]. ^•^NO can be enzymatically scavenged through Nitric Oxide Dioxygenase (NOD) or Nitric Oxide Reductases (NOR) [75] (S2 Table), and may also react non-enzymatically with reduced glutathione (GSH) to form S-nitrosoglutathione (GSNO) [21, 76]. Most cellular damage mediated by ^•^NO is attributed to the reaction of ^•^NO with O_2_^•−^ to produce Peroxinitrite (ONOO^−^) but this reaction is limited by the low extracellular concentration of ^•^NO in seawater [16].

Other important ROS, Singlet Oxygen (^1^O_2_), Peroxynitrite (ONOO^-^) and HO^•^ are not known to be directly produced nor scavenged by enzymatic processes [58, 77–81]. Because of the high reactivity of HO^•^, it is unlikely that there are any scavengers dedicated to HO^•^ specifically [58], although reactions with dissolved organic matter non-specifically scavenge extracellular HO^•^ [82].

### The black queen hypothesis

The Black Queen Hypothesis states that loss of function mutations may proceed so long as some interacting community members retain the function, and the function can occur outside a given cell [83]. The Black Queen Hypothesis was formulated on the basis of *Prochlorococcus*, which lost the genes encoding enzymes which scavenge H_2_O_2_. Instead, *Prochlorococcus* allows H_2_O_2_ outwards across the cell membrane to be dealt with by community members retaining the capacity to scavenge H_2_O_2_, thus saving *Prochlorococcus* the cost of maintaining the genes and metabolism for scavenging H_2_O_2_ [83, 84]. Growth and survival of *Prochlorococcus* indeed improves when co-cultured with ‘helper’ bacteria which carry genes for catalase [84–87].

### Hypotheses and significance

Given that ROS show differential abilities to cross cell membranes, and have widely different diffusion distances before destruction [88], we sought to study whether cell radius, colony formation, flagella, or diatom cell shape influence genomic allocations to ROS production and scavenging across diverse marine phytoplankters.

Hypothesis 1 **Cell radius across phytoplankton taxa does not influence the fraction of total gene content encoding O**_**2**_^**•−**^ **production, nor scavenging.** O_2_^•−^ is highly toxic and does not readily cross biological membranes [17], so diffusional losses of O_2_^•−^ from cells are limited, and cells need to retain capacity for detoxification of O_2_^•−^ across cell sizes.

Hypothesis 2 **Large phytoplankton allocate a smaller fraction of their total gene content to H**_**2**_**O**_**2**_ **and** ^**•**^**NO production and a larger fraction of their total gene content to H**_**2**_**O**_**2**_ **and** ^**•**^**NO scavenging.** H_2_O_2_ and ^•^NO have relatively low reactivity, with long intracellular and extracellular lifetimes leading to long potential diffusion paths before destruction. Both H_2_O_2_ and ^•^NO are uncharged and readily cross cell membranes (Table 1). Large cells have longer intracellular diffusional paths and a lower surface to volume ratios than do smaller cells [1]. Large cells are thus less prone to diffusional losses of intracellular H_2_O_2_ and ^•^NO. To maintain H_2_O_2_ and ^•^NO homeostasis in the face of slower diffusional losses of H_2_O_2_ or ^•^NO out of the cells to the environment, large phytoplankton may have a smaller fraction of their gene contents encoding H_2_O_2_ and ^•^NO production. In contrast, loss of function mutations on enzymes that scavenge H_2_O_2_ and ^•^NO would be more deleterious in large cells than in smaller cells [83, 84].

Hypothesis 3 **Flagellated phytoplankton have a larger fraction of their total gene content encoding H**_**2**_**O**_**2**_ **and** ^**•**^**NO production, and a smaller fraction of their total gene content encoding H**_**2**_**O**_**2**_ **and** ^**•**^**NO scavenging.** Increased motility in flagellated cells allows movement away from cytotoxic levels of H_2_O_2_ and ^•^NO, possibly complementing scavenging.

Hypothesis 4 **Colony forming phytoplankton have a smaller fraction of their total gene content encoding H**_**2**_**O**_**2**_ **and** ^**•**^**NO production, and a larger fraction of their total gene content encoding H**_**2**_**O**_**2**_ **and** ^**•**^**NO scavenging.** Cell spacing in colony forming phytoplankton is so small that the diffusional spheres of H_2_O_2_ or ^•^NO diffusing outwards from cells overlap with nearby cells [88], thereby shifting the requirements to maintain homeostasis within cells of a colony.

Hypothesis 5 **Pennate Diatoms allocate a larger fraction of their total gene content to H**_**2**_**O**_**2**_ **and** ^**•**^**NO production, and a smaller fraction of their total gene content to H**_**2**_**O**_**2**_ **and** ^**•**^**NO scavenging than do Centric Diatoms.** Pennate diatoms have a small minimum radii even at large biovolumes due to their elongated shape [89]. This cell shape of pennate diatoms allows for more diffusion of H_2_O_2_ and ^•^NO across the cell membrane due to the shorter mean diffusion paths to the cell surface and high surface area to volume ratio. To maintain homeostasis, pennate diatoms may have a larger fraction of their total gene content for H_2_O_2_ and ^•^NO production compared to centric diatoms. In contrast, pennate diatoms may have a smaller fraction of their gene content for H_2_O_2_ and ^•^NO scavenging, compared to centric diatoms.

Our work analyzed the fraction of the total genes in a genome or transcriptome associated with the metabolism of a particular ROS. The presence or absence of genes encoding specific ROS metabolizing enzymes may be caused by genetic drift, or may relate to a selective advantage linked to other metabolites of the same enzyme, rather than an enzymatic role in ROS metabolism, *per se*. Furthermore, the presence of a gene in a genome does not necessarily mean the encoded enzyme will be active, and closely related enzymes may mediate different activities in different organisms. The influence of non-enzymatic pathways such as carotenoids or tocopherols [42, 90, 91] likely affect the hypotheses listed above, but were beyond the frame of this study.

## Methods

### Data dictionary

S1 Table contains a data dictionary of variable names used in our analysis, their definitions and locations in code and data objects.

### Bioinformatic pipeline

**Fig 1:**
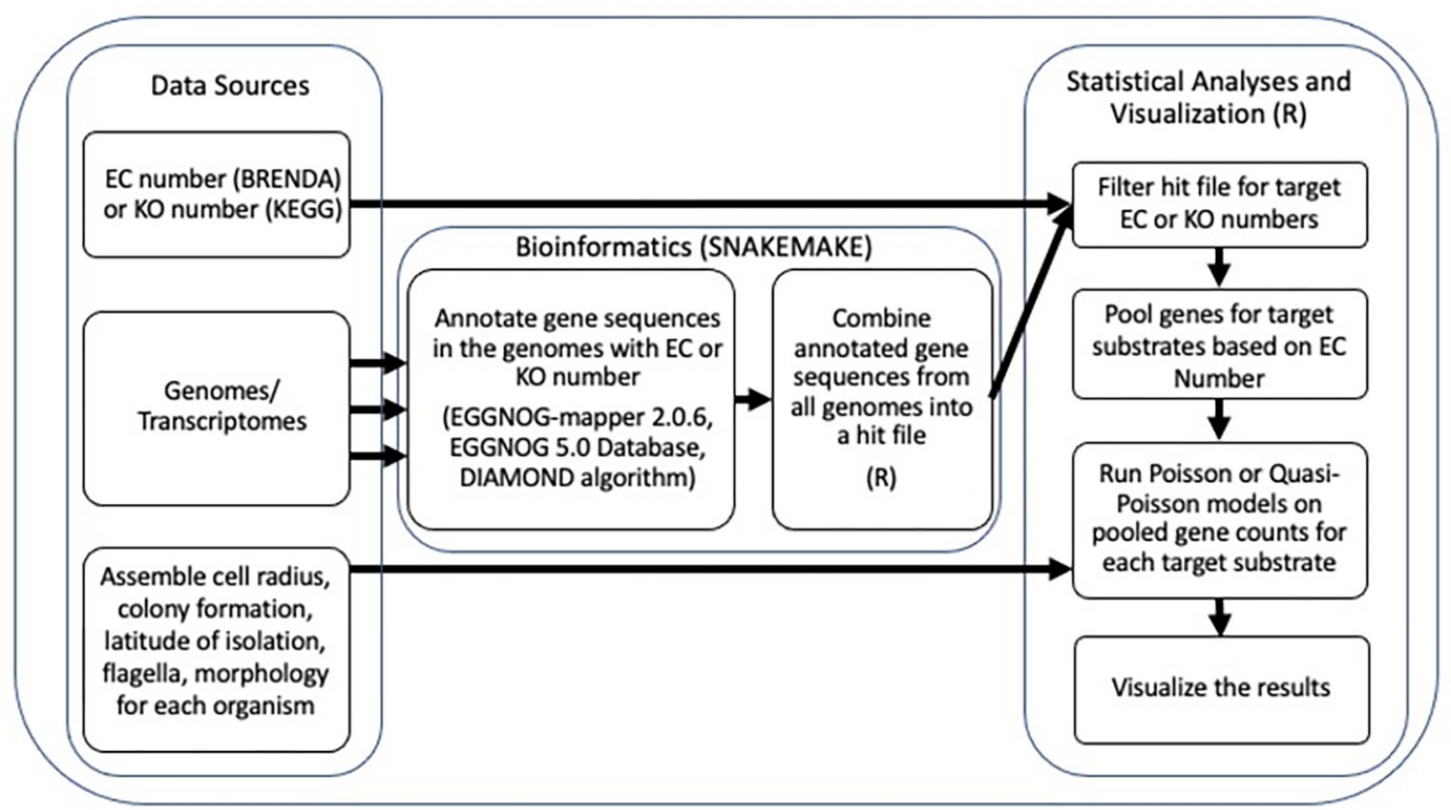
Summary flowchart of methods.

We downloaded Genomes and/or Transcriptomes of 146 diverse marine phytoplankton (S3 Table) from the National Center for Biotechnology Information (NCBI) [92]; Joint Genome Institute (JGI) [93, 94]; iMicrobe [95], European Nucleotide Archive (ENA) [96]; pico-PLAZA [97], 1000 Plants (1KP) [98]; and the Reef Genomics Database [99] (Fig 1).

We implemented an automated pipeline using Snakemake [100] to pass gene sequences from downloaded genomes or transciptomes, in.fasta format, to eggNOG-Mapper 2.0.6 [101, 102] and then used the DIAMOND algorithm [103] and the eggNOG 5.0 database [104], to annotate potential orthologs in each analyzed genome or transcriptome, using the following parameters: seed_ortholog_evalue = 0.001, seed_ortholog_score = 60, tax_scope = “auto,” go_evidence = “non-electronic,” query_cover = 20 and subject_cover = 0. The annotation generated for each gene model included (when available): the name of the matching ortholog (coded by eggNOG as ‘seed_eggNOG_ortholog’); E-value (coded by eggNOG as ‘seed_ortholog_evalue’); Score (coded by eggNOG as ‘seed_ortholog_score’); EC number (coded by eggNOG as ‘EC’); Kegg Orthology (KO) number (coded by eggNOG as ‘KEGG_ko’); Kegg Pathway (coded by eggNOG as ‘KEGG_Pathway’); Kegg Module (coded by eggNOG as ‘KEGG_Module’); Kegg Reaction (coded by eggNOG as ‘KEGG_Reaction’); Kegg Reaction Class (coded by eggNOG as ‘KEGG_rclass’); the predicted protein family (coded by eggNOG as ‘PFAMs’); Gene Ontology (GO) annotation (coded by eggNOG as ‘Gos’); as well as a description from eggNOG of the source organism of the matching ortholog (coded by eggNOG as ‘best_og_desc’). Note that comparison of sequences to the eggNOG 5.0 database generates non-supervised orthology annotations, and is subject to error if the underlying eggNOG annotation was inaccurate, or for functionally divergent orthologous gene sequences. The output of automatically annotated orthologs, from each genome or transcriptome, from the bioinformatic pipeline was compiled into one file CombinedHits.csv (to be submitted to the DRYAD database to support alternate analyses) (Fig 1).

### Overview of analysis of annotated genes

CombinedHits.csv was imported into a data frame (coded as ‘CombinedHits’) for analysis using R [105] running under RStudio [106], using the ‘tidyverse’ [107], ‘broom’ [108], ‘magrittr’ [109], ‘dplyr’ [110], ‘rcompanion’ [111], ‘gmodels’ [112], ‘stats’ [105], ‘AER’ [113] and ‘smatr’ [114] packages, and the ‘logit2prob’ function [115]. Graphics and tables were generated using the ‘ggplot2’ [116], ‘cowplot’ [117], ‘glue’ [118], ‘kableExtra’ [119], ‘corrplot’ [120], ‘ggfortify’ [121, 122], and ‘ggforce’ [123] packages (Fig 1). Formatted outputs were generated from Rmarkdown files using the ‘knitr’ [124–126] and ‘bookdown’ [127] packages.

In parallel we assembled metadata from the literature and culture collection databases for each phytoplankter for which we obtained a genome or transcriptome; including the cell radii in μm from 100% of organisms (S1 Fig); colony formation for 84% of organisms; cell shape from diatoms from 100% of diatoms; presence or absence of flagella as an index of potential motility from 100% of organisms; the genome size from all genomes; the total number of predicted gene models from 97% of organisms; and the total number of nuclear genes encoding ribosomal components from 100% of organisms (S3 Table); all stored in CellGenomeMetrics.csv (submitted to the DRYAD database to support alternate analyses; doi.org/10.5061/dryad.kh1893284) (Fig 1). For organisms for which only transcriptomes were available, we only included datasets for which the total number of detected different transcripts was available, as a proxy for the total number of predicted genes. Strains of brackish origin were included but we did not include obligate freshwater strains in our analyses.

Citations were managed using the Zotero (www.zotero.org) open access reference manager connected to Rstudio using the ‘citr’ [128] package. The Zotero library of citations for this paper is available at (https://www.zotero.org/groups/2333131/ros_phytoplankton).

We compared the Enzyme Commission Number (EC number) from CombinedHits to the BRENDA enzyme database [129] to identify enzymes annotated by BRENDA as ‘natural product’ or ‘natural substrate’ for H_2_O_2_, O_2_^•−^ or ^•^NO *in vivo* (S2 Table; Fig 1). We then used the EC Number to filter ‘CombinedHits’ to generate a subset containing only those orthologs encoding enzymes directly mediating metabolism, Production or Scavenging, of H_2_O_2_, O_2_^•−^ and ^•^NO.

From the ‘CombinedHits’ data frame, we filtered out some enzymes where the BRENDA annotations of ‘natural product’ or ‘natural substrate’ was questionable, in particular:

- Superoxide oxidase (EC:1.10.3.17) carries a BRENDA annotation of ‘natural product’ for O_2_^•−^, despite the BRENDA citation stating that O_2_^•−^ production from superoxide oxidase was only documented *in vitro* with an excess of ubiquinone [130].
- D-amino-acid oxidase was removed from counts of genes encoding H_2_O_2_ production, as the enzyme does not produce H_2_O_2_ *in vivo* [131].
- Bacterial non heme ferritin is listed under H_2_O_2_ production and scavenging as it produces H_2_O_2_ in the first of a two-step reaction and scavenges H_2_O_2_ in the second step [132].

From the subset of ‘CombinedHits’ of enzymes annotated for ROS metabolism, we grouped orthologs together by EC number and their Kegg Orthology number (KO number) and determined the occurrences of individual orthologs encoding each EC number, or KO number when EC number was not available, in a given organism. We merged this data subset with CellGenomeMetrics.csv to generate a dataset of genes encoding ROS metabolizing enzymes, as defined by the EC or KO number, along with characteristics of the source organism, combined into ‘MergedData.’ From the ‘CombinedHits’ data frame, we extracted and counted all genes annotated by eggNOG as ribosomal (Genes with the GO annotation ‘GO:0005840‘; coded as Ribosome_count), which we subsequently use as a proxy for housekeeping genes.

H_2_O_2_, O_2_^•−^ and ^•^NO differ in reactivity, stability, diffusion distance, effects on biomolecules and roles in cell signaling (Table 1). We therefore generated the total gene counts coding for the production or scavenging of each different ROS in a given organism, which were used to generate Poisson or Quasi-Poisson regressions (Fig 1). For ^•^NO, we also ran Binomial probability models to infer the cell size at which organism has an equal probability of having (or not having) the genomic capacity to encode nitric oxide production or scavenging. These presence/absence analyses were not run for H_2_O_2_ and O_2_^•−^ as all eukaryotic organisms either ubiquitously had the genomic capacity to scavenge H_2_O_2_ and O_2_^•−^; and to produce H_2_O_2_; whereas no organism had specific genomic capacity to produce O_2_^•−^.

### Data validation & justification of statistical analyses

Data from both genomes and transcriptomes were used in this analysis to gain wider representation from more taxa (S1 Fig). Data from the taxa with the largest radii were derived wholly from transcriptomes. Aside from the prokaryote genomes, sourced solely from within the 45° north south latitude band, the sampled phytoplankton did not exhibit taxonomic biases in source latitude of isolation, but were primarily coastal (S2 Fig). For 40 organisms we had both genomic and transcriptomic data, which we used to test assumptions on data distributions (S3 Fig). As expected, data coverage from paired genomes and transcriptomes derived from the same organism correlated well. Therefore, both genomic and transcriptomic data were available from the same organism, we used genomic data in subsequent analyses (S3 Table), but we used data from transcriptomes when genomes were not available. We validated the gene annotations generated by the snakemake bioinformatic pipeline by comparing the total number of genes encoding ROS metabolism data from a subset of ‘CombinedHits.csv’ to the total number of genes encoding ROS metabolism data from a manually annotated dataset generated during a pilot project (S3 Fig) [133, 134].

As expected, S4 Fig shows a strong correlation (Correlation of 0.87, p = 1.6×10^−49^) between manually generated ‘ROSGene_count’ and the automated ‘ROSGene_count’ from the snakemake pipeline.

S5 Fig shows that the frequencies of counts of genes encoding the metabolism of O_2_^•−^, H_2_O_2_ or ^•^NO within an organism are not normally distributed (Shapiro-Wilk Test [135] with a p-value of 6.4×10^−30^ for O_2_^•−^ scavenging, 9.4×10^−24^ for H_2_O_2_ production, 5×10^−25^ for H_2_O_2_ scavenging, 1.2×10^−18^ for ^•^NO production and 1.5×10^−30^ for ^•^NO scavenging). The frequencies of gene counts instead follow Poisson distributions. Therefore, for subsequent analyses we used Poisson or Quasi-Poisson regressions to compare the counts of genes that encode the production or scavenging of O_2_^•−^, H_2_O_2_ or ^•^NO within an organism to log_10_ of the median cell radius in μm. Code used to produce the Poisson and Quasi-Poisson models is on https://github.com/FundyPhytoPhys/ROS_bioinfo/tree/master/ROSGenomicPatternsAcrossMarinePhytoplankton.

Quasi-Poisson regressions were used when the Poisson regression was over-dispersed (dispersion > 1, p < 0.05) as determined by the ‘AER’ package [113]. A Poisson regression followed by a chi-squared test, or a Quasi-Poisson regression followed by an F-test, was used to obtain p-values [136], with an alpha value of ≤0.05 as the threshold for statistical significance of regressions; and a pseudo-R^2^ was calculated using the McFadden R^2^ method [137].

**Fig 2:**
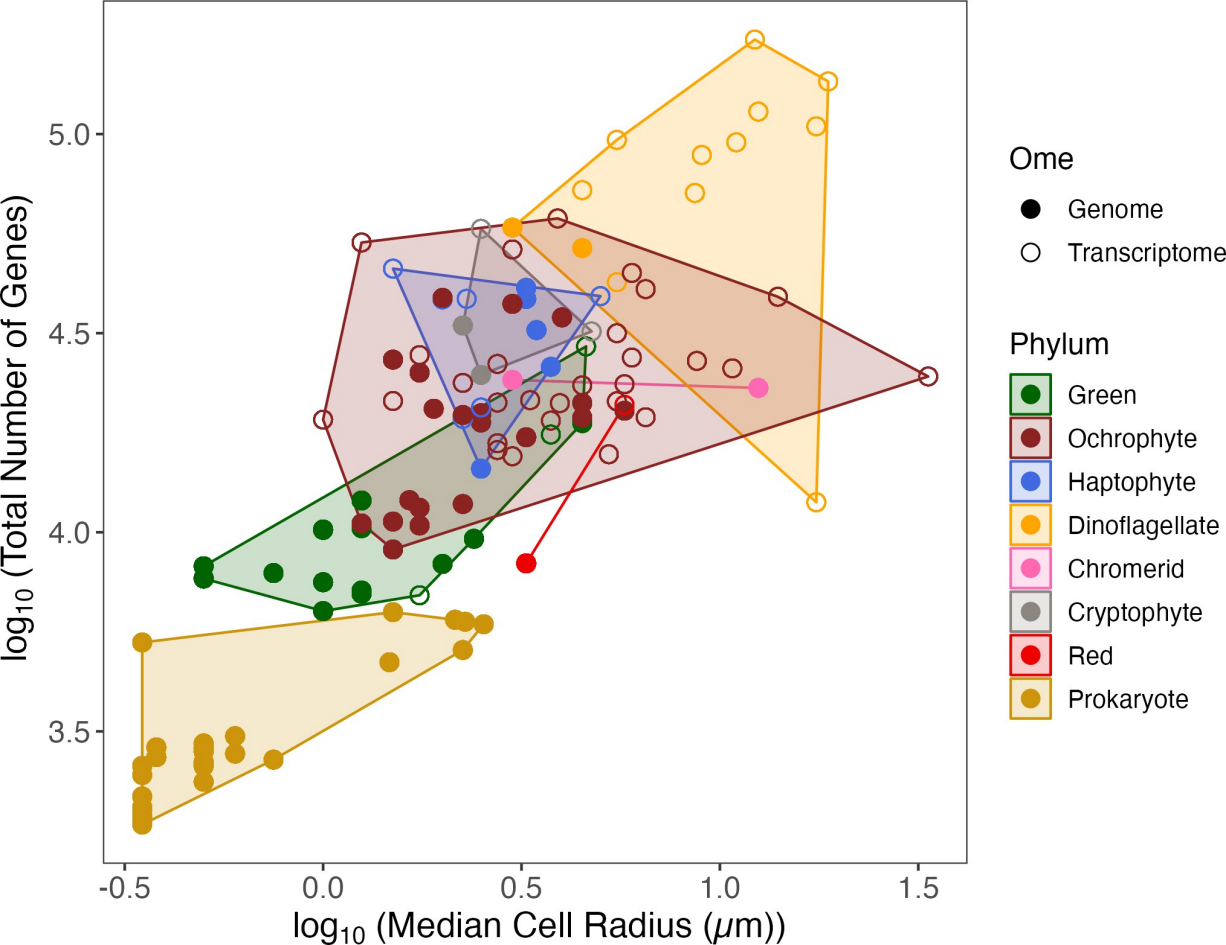
Comparison of log_10_ of the total number of genes in an organism (‘log_GeneModels_count’) to log_10_ of the median cell radius in μm (‘log_Radius_um’). Colour corresponds to the taxonomic lineage (‘Phylum’), whereas symbol shape corresponds to the source of the data, whether Genome or Transcriptome (‘Ome’). Citations for data sources are in S3 Table.

The total number of genes in each organism increased with the median cell radius, and also varied among the taxonomic lineages (coded as ‘Phylum’) (Fig 2). Taxonomic lineage, in turn, interacts strongly with the median cell radius. For our analyses, we sought to detect effects of cell radius upon the fraction of total genes encoding ROS metabolism. We therefore included an offset of the total number of genes in the organism in the Poisson or Quasi-Poisson regressions, which is equivalent to normalizing the number of genes encoding the production or scavenging of H_2_O_2_, O_2_^•−^ or ^•^NO, to the total number of genes in the organism (‘GeneModel_count’). We thereby offset the general increase in ‘GeneModel_count’ with increasing the median cell radius. Because of the strong interaction between the median cell radius and taxonomic lineage (S1 Fig), we did not include Phylum as a co-variate in our subsequent regressions of normalized gene counts vs. median cell radius. Thus, we did not analyze specific influences of Phylum upon gene counts for ROS metabolism. Poisson or Quasi-Poisson regressions were run both with or without ‘Colony’ and ‘Flagella’ as co-variates.

The total number of ribosomal genes did not increase with median cell radius, but did vary with taxa (S6 Fig). Therefore, we also normalized the number of genes encoding the production or scavenging of H_2_O_2_, O_2_^•−^ or ^•^NO, to the total number of ribosomal genes in the organism (‘Ribosome_count’), as a proxy for housekeeping genes. Because median cell radius and taxonomic lineage did not interact in this plot, we included Phylum, or ‘Colony’ and ‘Flagella,’ as co-variates in our Poisson or Quasi-Poisson regressions of normalized ribosomal gene counts vs. median cell radius.

To further investigate possible influences of colony formation, the presence of flagella or diatom cell shape (pennate or centric), independent of cell size, upon the fraction of genes that encode the metabolism of H_2_O_2_, O_2_^•−^ or ^•^NO, we used a Wilcoxon test [138] after binning data across all diatom sizes.

## Results and discussion

### Superoxide

Although there are enzymes that specifically produce O_2_^•−^ [139], in the marine phytoplankton genomes and transcriptomes that we analyzed, we did not detect any genes that encode for such enzymes (S2 Table), based on the BRENDA annotation. It is however worth noting the presence of genes annotated as encoding NADPH Oxidase (NOX) in some phytoplankton genomes. NOX can produce either H_2_O_2_ or O_2_^•−^ depending on the NOX isoform. NOX is included in our analyses as a H_2_O_2_ producer, in accordance with the BRENDA annotation of the enzyme (S2 Table). Further analyses of the detected NOX isoforms might identify whether they include isoforms that produce O_2_^•−^. Sequences that are similar to Glutathione Reductase (GR) have been documented to produce enzymes that produce extracellular O_2_^•−^ in the diatom *Thalassiosira oceanica* [139]. We found sequences annotated as GR across all phytoplankton genomes (S4 Table), which likely include genes encoding enzymes producing O_2_^•−^. Phytoplankton may need to maintain working extracellular concentrations of O_2_^•−^, since decreasing the extracellular concentration of O_2_^•−^ can hinder cell growth [48]. [48] further explains that the downregulation of Superoxide Dismutase (SOD, EC:1.15.1.1) genes at peak light levels by *Prochlorococcus* [140] may allow *Prochlorococcus* to maintain ‘working levels’ of extracellular O_2_^•−^. Beyond putative enzymatically mediated production of O_2_^•−^, non-enzymatic processes associated with cells can also produce O_2_^•−^ to variable extents, notably from side-reactions of electron transport [38, 141, 142] particularly under stress conditions.

Given that the O_2_^•−^ is poorly diffusible across membranes, intracellularly produced O_2_^•−^ has to be scavenged to limit detrimental reactions of O_2_^•−^ [143]. As a result, the analyzed phytoplankton universally maintain genomic capacity encoding the ubiquitous O_2_^•−^ scavenging enzyme SOD (S8 Fig), with the exception of a single transcriptome from *Micromonas polaris*. Genes annotated as encoding the enzyme Superoxide Oxidase (SOO, EC:1.10.3.17) were present in a few diatom species (*Leptocylindrus danicus*, *Chaetoceros curvicetus* and *Thalassiosira minuscula* CCMP1093) and prokaryotes (*Crocosphaera* spp.). Genes encoding the enzyme Superoxide Reductase (SOR, EC:1.15.1.2) were detected in some diatoms (*Pseudo-nitzschia fradulenta* WWA7 and *Seminavis robusta* D6), and in the haptophyte *Pleurochrysis carterae* CCMP456. BLAST searches support these annotations of genes for SOO and SOR in the genomes of some phytoplankters. These results should be confirmed by enzyme assays, to identify if the genes indeed encode active enzymes. Finer trends in genomic allocations to O_2_^•−^ scavenging may emerge among the metallo-forms of SOD [144]. For example, in pilot runs discriminating among SOD metallo-forms we found that pico-prasinophytes encode Mn-SOD instead of the Fe-SOD encoded in genomes from larger green algal phytoplankters (Data not visualized) [133].

**Fig 3:**
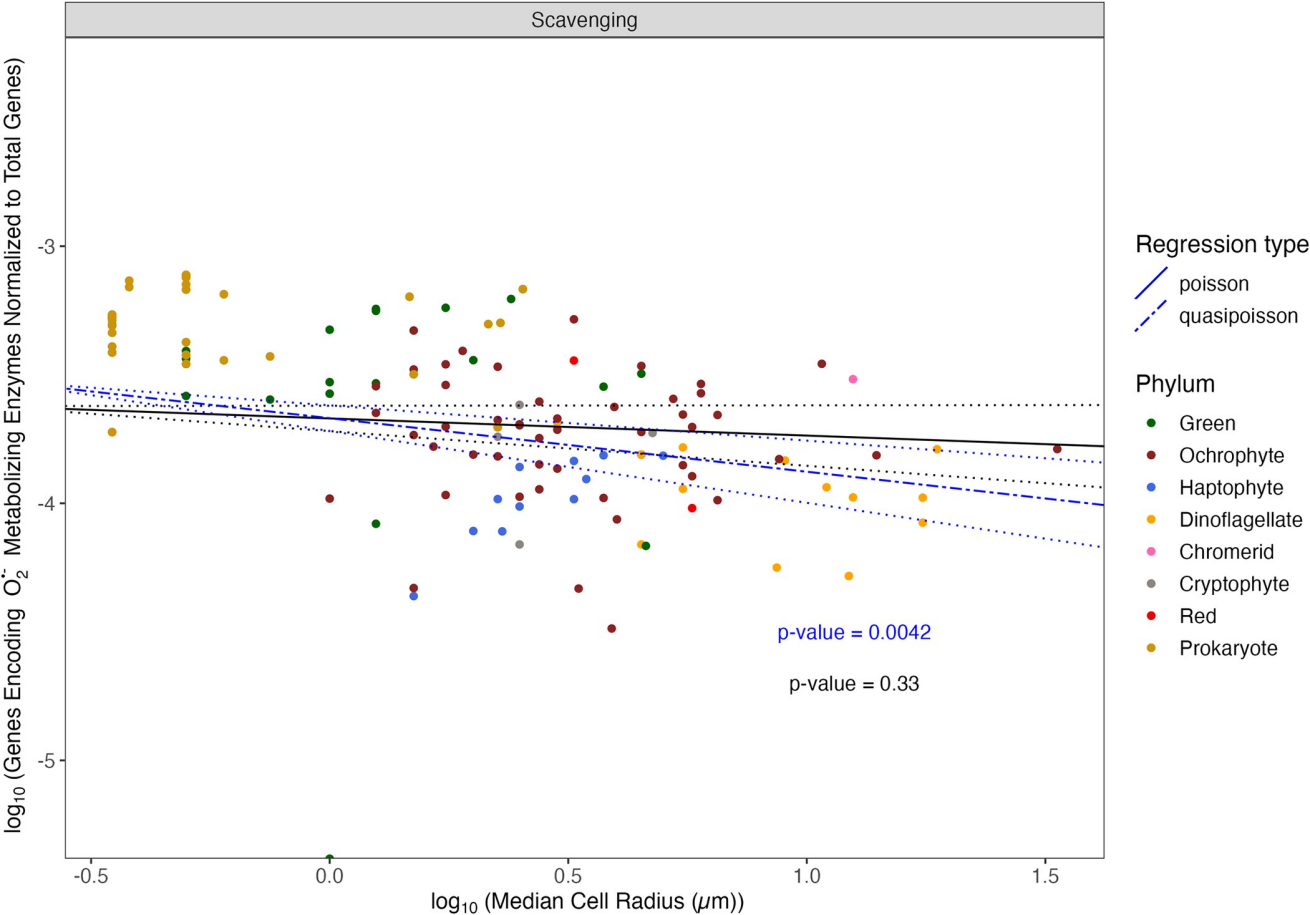
Comparison of log_10_ (Total number of genes encoding O2•− metabolizing enzymes (‘SupOx_count’) normalized to the total number of genes present in each organism (‘GeneModels_count’)) vs. the log_10_ (median cell radius in μm (‘log_Radius_um’)). Poisson (solid line) or Quasi-Poisson (dashed line) regressions fitted to data ± Standard Error (dotted line). Regressions were run with (black line) or without (blue line) ‘Colony’ and ‘Flagella’ as co-variates. Selected prokaryote genomes are presented for comparison, but excluded from the presented regressions. Symbol color corresponds to taxon lineage (‘Phylum’).

With increasing cell radius, eukaryotic phytoplankton have a smaller fraction of their total genes encoding scavenging of O_2_^•−^ (Fig 3, Blue line, Slope = -2.1×10^−1^ ± 7.1×10^−2^, p-value = 4.2×10^−3^, pseudo-R^2^ = 0.087). The negative slope does not support our Hypothesis 1 that phytoplankton do not differentially allocate a changing fraction of their total gene content to O_2_^•−^ scavenging with increasing cell size. Including ‘Flagella’ and ‘Colony’ as co-variates in the regression results, however, in a slope that is not statistically different from zero (Fig 3, Black line, Slope = -6.7×10^−2^ ± 6.8×10^−2^, p-value = 3.3×10^−1^), driven by the influence of ‘Flagella’ (p-value = 3.7×10^−2^) but not ‘Colony’ (p-value = 8.6×10^−1^). O_2_^•−^ metabolism in phytoplankton appears to be mediated by a nearly fixed set of core genes that do not change with increasing total gene count, thus the fractional gene allocation to O_2_^•−^ decreases as cell radius, and the co-varying total gene count increases. Therefore, gene dosage does not emerge as a factor in phytoplankton O_2_^•−^ metabolism. With increasing cell radius, eukaryotic phytoplankton have no cell-size associated difference in genes encoding superoxide scavenging, when normalized to total ribosomal genes, suggesting that as a fraction of housekeeping genes, cells do not increase their genomic capacity to scavenge superoxide, consistent with our hypothesis 1 (Data not visualized, Slope = -2.4×10^−1^ ± 2.5×10^−1^, p-value = 3.3×10^−1^).

**Fig 4:**
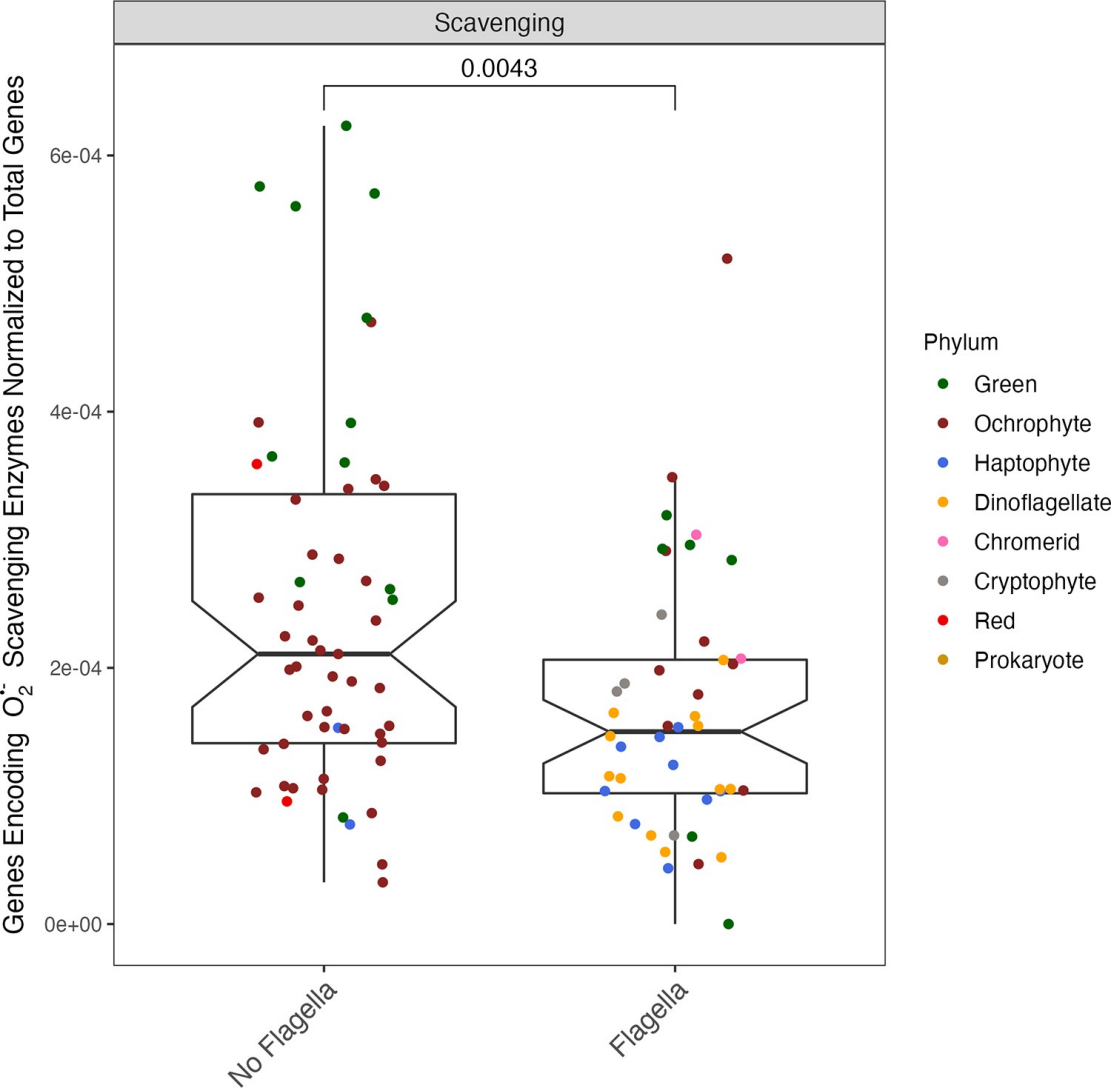
Comparison of total number of genes encoding O2•− scavenging enzymes (‘SupOx_count’) normalized to the total number of genes present in each organism (‘GeneModels_count’)) vs. the presence or absence of flagella in the organism. Symbol color corresponds to taxon lineage (‘Phylum’). Notch spans ± standard error of the median. Box spans median ± 1 quartile of the data. Whiskers span the range excluding outliers in the data. Citations for data sources can be found in S3 Table.

Consistent with the significant influence of flagella on the regressions vs. median cell radius (Fig 3), flagellated phytoplankton, irrespective of size, have a smaller proportion of their total gene content encoding O_2_^•−^ scavenging (Fig 4, p-value = 4.3×10^−3^), than do non-flagellated phytoplankton. Similarly, irrespective of size, flagellated phytoplankton have a smaller ratio of genes encoding O_2_^•−^ scavenging to their total ribosomal genes (Data not visualized, p-value = 3.2×10^−10^). This suggests that cellular motility contributes to phytoplankton homeostasis of O_2_^•−^, possibly by supporting escape from localized extracellular pockets of O_2_^•−^. This decrease in proportional allocation to O_2_^•−^ scavenging is also arithmetically consistent with the difference in the number of total genes between flagellated and non-flagellated phytoplankton, whereby flagellated phytoplankton have more total genes.

Pennate and centric diatoms have similar fractions of their genomes encoding O_2_^•−^ scavenging (p-value = 9.7×10^−1^), as well as similar ratios of O_2_^•−^ scavenging genes to their housekeeping ribosomal genes (p-value = 8.1×10^−1^) (Data not visualized). Our results support our hypothesis that differential diffusional exchange across diatoms of different shape does not influence the fraction of total gene content encoding O_2_^•−^ scavenging enzymes, because O_2_^•−^ diffusion outwards is limited by the cell membrane, regardless of cell shape (Hypothesis 1). Differences between genomic patterns of pennate and centric diatoms may arise when comparing metallo-forms of SOD, noting that [145] found that pennate diatoms transcribe Cu/Zn-SOD but not Fe-SOD, whereas centric diatoms transcribe Fe-SOD more frequently than they transcribe Cu/Zn-SOD.

### Hydrogen peroxide

All prokaryotic (S9 Fig) and eukaryotic (S10 Fig) phytoplankton, with the exception of a single transcriptome from the prasinophyte *Micromonas polaris*, have genes encoding H_2_O_2_ producing enzymes, as they all carry gene(s) encoding the ubiquitous enzyme Superoxide Dismutase. Genes encoding oxidases producing H_2_O_2_ include copropophyrinogen oxidase, found across all eukaryotic and prokaryotic phytoplankton, with the exception of one transcriptome. Genes encoding thiol oxidase and acyl CoA oxidase are also found in nearly all eukaryotic phytoplankton, with the exceptions of three transcriptomes. Genes encoding L-aspartate oxidase are found in nearly all prokaryotes, and all green algae, but are nearly absent from other eukaryotic taxa. Sarcosine oxidase is not present in small diatoms and small green algae, but is present in nearly all dinoflagellates and haptophytes. (S)-2-hydroxy-acid oxidase (whose EC number includes glycolate oxidase) is found in most eukaryotic phytoplankton, but rarely in dinoflagellates.

Most prokaryotic phytoplankton (S9 Fig) and all eukaryotic (S10 Fig), have genes encoding H_2_O_2_ scavenging enzymes. Some strains of *Prochlorococcus* and *Synechococcus* have lost all genomic capacity to scavenge H_2_O_2_, and appear to rely on co-occurring hosts for H_2_O_2_ scavenging [83, 84, 87].

The absence of catalase from most analyzed cyanobacterial genomes supports [146] who analyzed 44 different cyanobacterial genomes and found that only *Nostoc punctiforme* PCC73102 retained a full gene encoding catalase. In our analyses, only *Synechococcus elongatus* PCC11802 maintained a catalase encoding gene (S9 Fig). In the greens, catalase has been lost from the smaller prasinophytes but is maintained in the larger greens (S10 Fig). The loss of catalase from smaller green algae may be evidence of the Black Queen Hypothesis in action [83], in that H_2_O_2_ can passively diffuse out of the smaller green algae but diffuses less out of larger green algae. Loss of function mutations in catalase encoding genes in small algae are therefore less deleterious than they would be to large green algae. Catalase, with a K_M_ of ~220 mM, may be poorly retained because the cells maintain some genomic capacity to scavenge H_2_O_2_ using the enzymes ascorbate peroxidase, glutathione peroxidase and Cytochrome C peroxidase (S10 Fig), with K_M_ in the low μM range [146].

Our results support an earlier suggestion that bigger genomic capacity for H_2_O_2_ scavenging in *Synechococcus* compared to *Prochlorococcus* is a result of the larger size in *Synechococcus* compared to *Prochlorococcus* [84] (S9 Fig). It is however important to note the vast differences between prokaryotic and eukaryotic phytoplankton, with most eukaryotic phytoplankton, regardless of lineage, maintaining the genomic capacity to produce ascorbate peroxidase, glutathione peroxidase and Cytochrome C peroxidase (S10 Fig). Peroxidases are involved in pathways beyond simple ROS scavenging, including the Halliwell-Asada cycle for ascorbate peroxidase [147]. *Ostreococcus*, the smallest prasinophyte has a radius of 0.5 μm, comparable to that of the prokaryote *Synechococcus* (S3 Table), and would therefore share a similarly short diffusion path length. Nevertheless *Ostreococcus*, in common with other eukaryotes, retains genomic capacities to produce ascorbate peroxidase, glutathione peroxidase and cytochrome c peroxidase, which may thus reflect the cost of being eukaryotic (S10 Fig).

**Fig 5:**
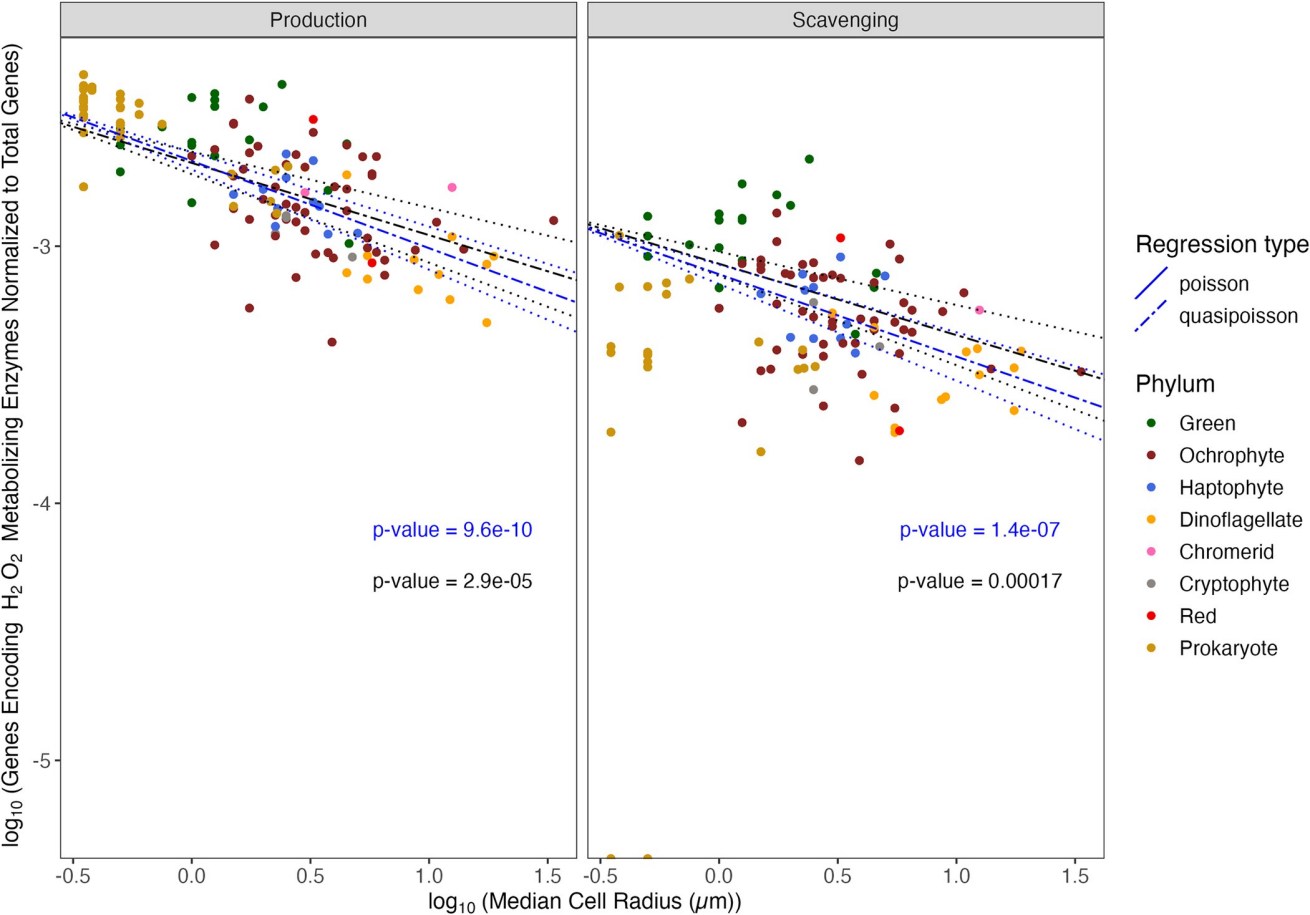
Comparison of log_10_ (Total number of genes encoding H_2_O_2_ metabolizing enzymes (‘HyPe_count’) normalized to the total number of genes present in each organism (‘GeneModels_count’)) vs. the log_10_ (median cell radius in μm (‘log_Radius_um’)). Poisson (solid line) or Quasi-Poisson (dashed line) regressions fitted to data ± Standard Error (dotted line). Regressions were run with (black line) or without (blue line) ‘Colony’ and ‘Flagella’ as co-variates. Selected prokaryote genomes are presented for comparison, but excluded from the presented regressions. Symbol color corresponds to taxon lineage (‘Phylum’). Citations for data sources are in S3 Table.

With increasing cell radius, eukaryotic phytoplankton have a smaller fraction of their total genes encoding the production of H_2_O_2_ (Fig 5, Blue line, Slope = -3.4×10^−1^ ± 5×10^−2^, p-value = 9.6×10^−10^, pseudo-R^2^ = 0.34). Including ‘Flagella’ and ‘Colony’ as co-variates did not significantly alter this pattern (Black line, ‘Flagella’ p-value = 8.4×10^−1^, ‘Colony’ p-value = 4.7×10^−1^). The pattern of a smaller fraction of total genes for H_2_O_2_ production with increasing cell radius supports our Hypothesis 2 that larger phytoplankton counter decreasing diffusional loss of H_2_O_2_ out of cells through a lower genomic capacity for H_2_O_2_ production, whereas losses of genes encoding H_2_O_2_ producing enzymes are more costly to small phytoplankton (Fig 5). [7] found that a major influence upon the capacity for production of H_2_O_2_ is whether or not the organism can form blooms, with bloom forming species producing more H_2_O_2_. The ability to form blooms was not analyzed in our data as we did not find systematic information on potentials for bloom formation across taxa.

With increasing cell radius, eukaryotic phytoplankton also have a smaller fraction of their total genes encoding the capacity to scavenge H_2_O_2_ (Fig 5, Blue line, Slope = -3.2×10^−1^ ± 5.6×10^−2^, p-value = 1.4×10^−7^, pseudo-R^2^ = 0.26). Including ‘Flagella’ and ‘Colony’ as co-variates did not influence the negative slope of the fraction of total genes encoding H_2_O_2_ scavenging with increasing median cell radius (Fig 5, Black line, ‘Flagella’ p-value = 4.1×10^−1^, ‘Colony’ p-value = 1.6×10^−1^). A parallel analysis focusing only on small phytoplankton such as pico-cyanobacteria and pico-prasinophytes might yield different results as more such genomes are sequenced, since [148] found that H_2_O_2_ added to seawater at a concentration of 1.6 mg L^-1^ did not affect cells with a radius larger than 1 to 1.5 μm, but differentially harmed the picoprasinophyte *Micromonas pusilla*.

Because median cell radius co-varied with Taxa, we generally excluded Taxa as a co-variate from our regressions, in order to focus on any cross-taxon patterns driven by changing median cell radius. Nevertheless, representatives of the Ochrophyte Phylum alone spanned more than an order of magnitude in median cell radius. We therefore tested whether the log_10_(total number of genes encoding the metabolism of O_2_^•−^, H_2_O_2_ or ^•^NO) varied with the log_10_ (median cell radius) across the Ochrophytes alone (S7 Fig). We found that across Ochrophytes, the fraction of total genes encoding the production of H_2_O_2_ decreased with increasing cell radius (Slope = -1.6×10^−1^ ± 9.4×10^−2^), although the p-value for the regression was only 1×10^−1^). This marginal decrease in the total number of genes encoding H_2_O_2_ production with increasing median cell radius in Ochrophytes again tends to support our Hypothesis 2, with data from within a single phylum to limit confounding influences of diverse evolutionary histories and cell biologies upon patterns.

H_2_O_2_ production (Slope = -3.7×10^−1^ ± 2×10^−1^, p-value = 6.7×10^−2^) and scavenging (Slope = -3.5×10^−1^ ± 2.1×10^−1^, p-value = 1×10^−1^) allocations were steady with increasing cell size, relative to the ribosomal housekeeping gene proxy. But, genes for H_2_O_2_ production and scavenging are diluted by increasing total gene counts with increasing cell size.

Pennate and centric diatoms do not show statistically significant differences in the fraction of their total gene content encoding the production (p-value = 1.9×10^−1^) nor the scavenging of H_2_O_2_ (p-value = 9.6×10^−2^). Pennate and centric diatoms also do not show statistically significant differences in the ratio of genes encoding production (p-value = 3.3×10^−1^) nor the scavenging of H_2_O_2_ (p-value = 3.9×10^−1^), normalized to their ribosomal gene content encoding H_2_O_2_. These results do not support our Hypothesis 5 that pennates have more genes encoding H_2_O_2_ producing enzymes due to their higher surface area to volume ratio (Data not visualized).

### Nitric oxide

In the genomes and transcriptomes that we analysed, Nitric Oxide Synthase (NOS, EC:1.14.13.39), although often absent, was the most frequently occurring ^•^NO producing enzyme encoded (S11 Fig), but was not encoded, or at least not annotated, among prokaryotic phytoplankton (Data not visualized).

Nitric Oxide Dioxygenase (NOD, EC:1.14.12.17) was the most frequently occurring of the ^•^NO scavenging enzymes (S11 Fig). NOD sequences were identified in some eukaryotes, but were either not annotated, or not present in *Prochlorococcus*, most green algae and most centric diatoms. A NOS-like sequence that also has Nitric Oxide Dioxygenase-like function [149] has recently been identified in *Synechococcus*, which might encode NOD activity in some strains lacking annotated NOD sequences.

**Fig 6:**
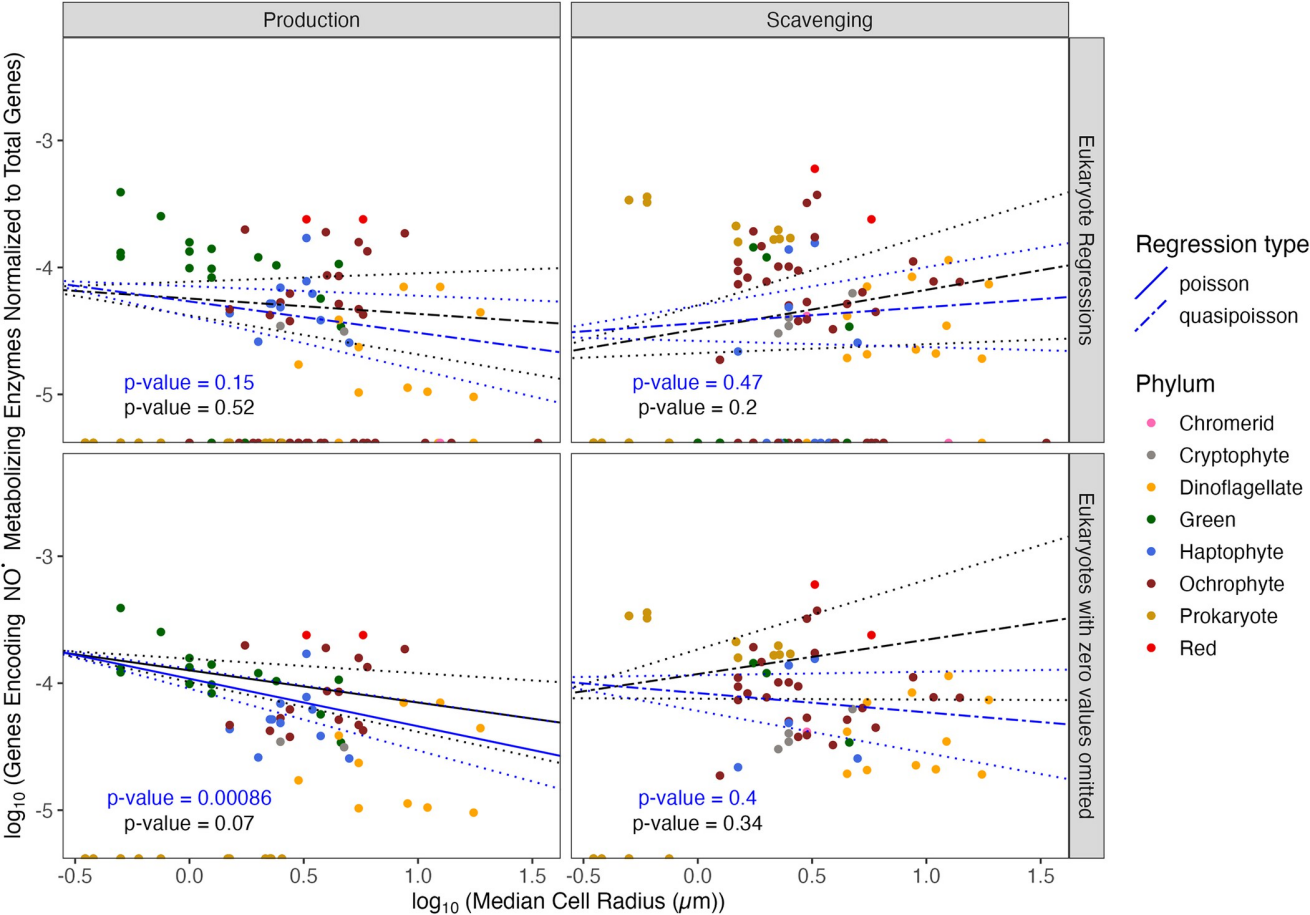
Comparison of log_10_ (Total number of genes encoding ^•^NO metabolizing enzymes (‘NitOx_count’) normalized to the total number of genes present in each organism (‘GeneModels_count’)) vs. the log_10_ (median cell radius in μm (‘log_Radius_um’)). Poisson (solid line) or Quasi-Poisson (dashed line) regressions fitted to data ± Standard Error (dotted line). Regressions were run with (black line) or without (blue line) ‘Colony’ and ‘Flagella’ as co-variates. Selected prokaryote genomes are presented for comparison, but excluded from the presented regressions. Symbol color corresponds to taxon lineage (‘Phylum’).

With increasing cell radius eukaryotic phytoplankton do not vary in the fraction of total genes encoding the capacity to produce ^•^NO (Fig 6, Blue line, Slope = -2.5×10^−1^ ± 1.7×10^−1^, p-value = 1.5×10^−1^). We re-ran the Quasi-Poisson, excluding those phytoplankton that completely lack genes encoding enzymes for ^•^NO production (NitOx_count = 0, points along the x-axis), which resulted in a decreasing slope with increasing cell radius. Thus, those phytoplankton with any detected capacity to produce ^•^NO indeed have a smaller fraction of their total genes encoding ^•^NO production with increasing radius (Fig 6, Blue line, Slope = -3.7×10^−1^ ± 1.1×10^−1^, p-value = 8.6×10^−4^, pseudo-R^2^ = 0.13). Including ‘Flagella’ and ‘Colony’ as co-factors for the regression that solely looks at phytoplankton with the genomic capacity to produce ^•^NO resulted in a slope that is no longer significantly different from zero (Fig 6, Black line, Slope = -2.5×10^−1^ ± 1.4×10^−1^, p-value = 7×10^−2^), driven by the influence of ‘Flagella’ (p-value = 1.4×10^−4^), but not ‘Colony’ (p-value = 1.8×10^−1^).

With increasing cell radius, eukaryotic phytoplankton do not vary in the fraction of their total genes encoding the capacity to scavenge ^•^NO, Quasi-Poisson regression slope not significantly different from zero (Fig 6, Blue line, Slope = 1.3×10^−1^ ± 1.8×10^−1^, p-value = 4.7×10^−1^).

Non-enzymatic paths contribute to intracellular and extracellular ^•^NO production [150], and may explain the absences of genes encoding ^•^NO production from some genomes across taxonomic lineages. Alternately, ^•^NO homeostasis may be achieved in some lineages by regulating the active cellular uptake and release of intracellular ^•^NO, as has been recently demonstrated in humans [151]. Although NOD sequences have only been identified from phytoplankton through meta-transcriptomic analyses, in diatoms, haptophytes and dinoflagellates [152], there is limited understanding as to what may contribute to the active removal of ^•^NO, and the lack of ^•^NO scavenging genes across multiple phytoplankters. More research is needed on possible contributions of NOD to the active removal of ^•^NO, as well as the NOS sequences detected in *Synechococcus* that also display NOD-like activity [149]. Perhaps the low toxicity of ^•^NO does not warrant the active removal of ^•^NO as long as the concentration does not exceed the toxic threshold. This explanation is plausible given that *Platymonas helgolandica*, *Platymonas subcordiformis*, *Skeletonema costatum*, *Gymnodinium* sp., and *Prorocentrum donghaiense* showed optimum growth in the presence of ^•^NO concentrations between 10^−9^ and 10^−6^ mol L^-1^ [153], which are higher than the concentrations found in the ocean (Table 1).

A binomial model comparing the presence or absence of genes that encode the production of ^•^NO shows no cell size effect (slope = -3.5×10^−1^, p = 1.4×10^−1^). Including ‘Flagella’ as a co-variate does not alter these results, but does show that flagellated phytoplankton have higher likelihood of presence of ^•^NO production than do non-flagellated phytoplankton (S12 Fig, p = 5.6×10^−5^). Including ‘Colony’ as a co-variate does not show a cell size effect, nor a difference in the likelihood of ^•^NO production between colony and non-colony forming phytoplankton (p = 1.3×10^−1^).

In contrast, larger phytoplankton are more likely to have a gene encoding ^•^NO scavenging (slope = 1.1×10^0^, p = 2×10^−4^). This trend is not influenced by flagella (p = 1.9×10^−1^) nor colony formation (p = 1.3×10^−1^). This pattern supports our hypothesis that larger, diffusionally limited cells, have a stronger requirement for ^•^NO scavenging (Hypothesis 2).

**Fig 7:**
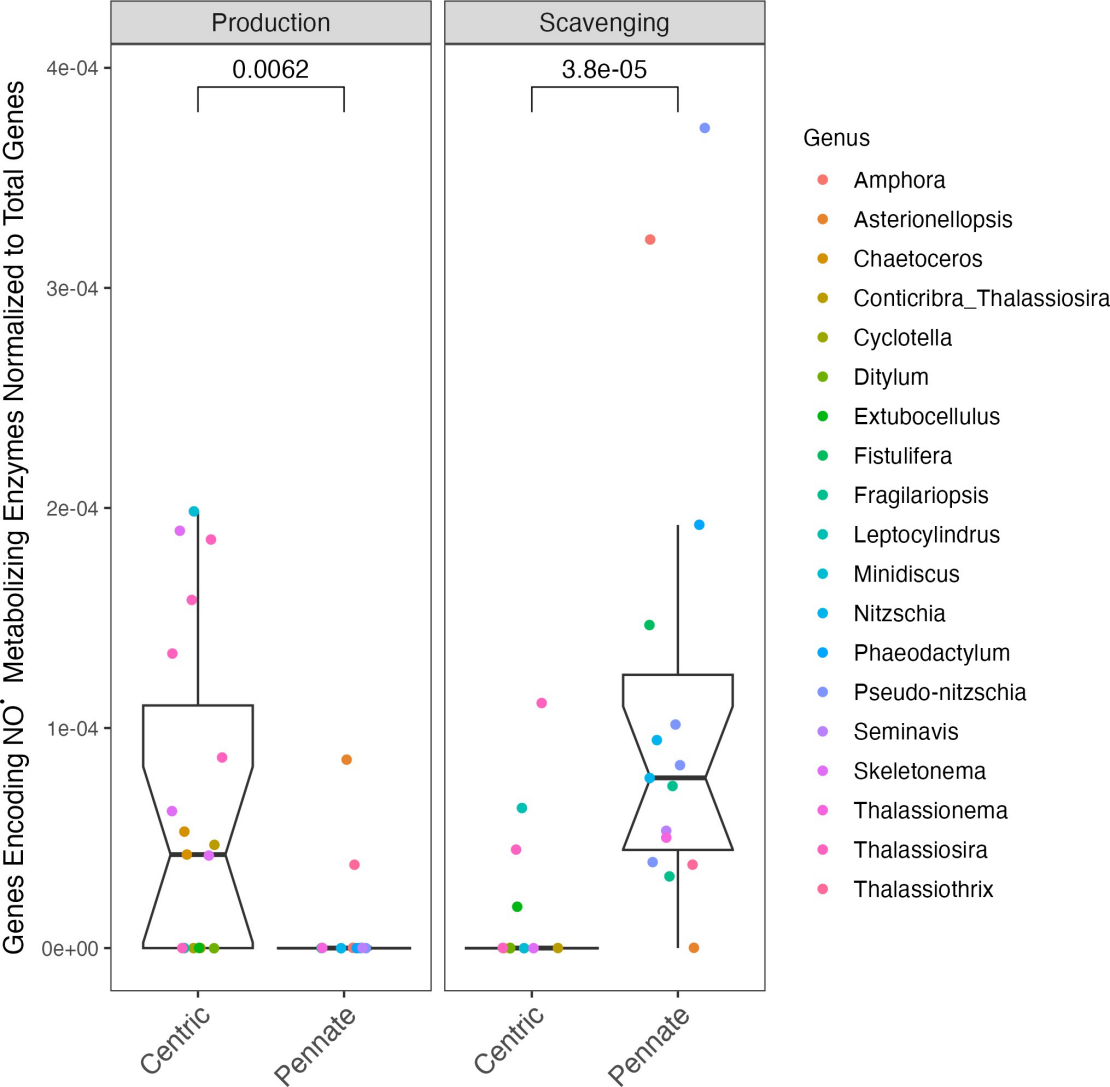
Comparison of total number of genes encoding ^•^NO metabolizing enzymes (‘NitOx_count’) normalized to the total number of genes present in each diatom (‘GeneModels_count’) vs. the growth form of the diatom (‘PennateCentric’). Symbol color corresponds to taxon lineage (‘Phylum’). Notch spans ± standard error of the median. Box spans median ± 1 quartile of the data. Whiskers span the range excluding outliers in the data. Citations for data sources can be found in S3 Table.

Most centric diatoms carry genes annotated as encoding ^•^NO producing enzymes, whereas most pennate diatoms do not (p-value = 6.2×10^−3^) when normalized to total genes, and when normalized to ribosomal genes (p-value = 1.1×10^−2^). In contrast, most centric diatoms lack genes annotated as encoding ^•^NO scavenging enzymes, whereas most pennate diatoms carry those genes (p-value = 3.8×10^−5^) when normalized to total genes, and p-value = 2.4×10^−5^ when normalized to ribosomal genes) (Fig 7).

The larger fractional gene allocation to ^•^NO production, and smaller fraction of genes that encode ^•^NO scavenging enzymes, in centric diatoms (Fig 7) counters our hypothesis that diffusion from pennate diatoms would drive gene allocations in favor of ^•^NO production (Hypothesis 5). Given the strong contrast in annotated ^•^NO metabolism genes, it is likely that ^•^NO has regulatory or signaling roles that vary systematically between pennate and centric diatoms, outside any diffusional influences. For example, ^•^NO inhibits diatom adhesion to substrate [72, 154]. Pennates are more likely to grow adhered in biofilms [155], which may explain the striking differences in total gene allocation to ^•^NO production and scavenging. Alternately, [156] identified putative NOS sequences in the transcriptomes of three pennate diatom species (*Pseudo-nitzschia arenysensis*, *Pseudo-nitzschia delicatissima* and *Pseudo-nitzschia multistriata*), so it is possible the apparent lack of ^•^NO producing sequences in pennates is due to errors in the unsupervised annotations from eggNOG.

### Summary

We analyzed the fractions of the total genes in the genome that are associated with the metabolisms of three major ROS. It is important to note that the content of genes encoding specific ROS metabolizing enzymes may be caused by genetic drift, or may relate to a selective advantage linked to other metabolites of the same enzymes, rather than an enzyme role in ROS metabolism, *per se*. Furthermore, the gene presence or gene count in a genome is only one influence on the potential activity of the encoded enzyme, and closely related enzymes may confer different activities in different organisms.

The differential reactivities, diffusion distances, diffusibilities across cell membranes, and roles in cell signaling of H_2_O_2_, O_2_^•−^ and ^•^NO (Table 1) influence genomic allocation patterns for the production and scavenging of these three distinct ROS.

O_2_^•−^ has high reactivity, short intracellular and extracellular lifetimes and limited cell membrane crossing. We did not find genes specifically encoding O_2_^•−^ production in eukaryotic phytoplankton genomes. As expected, genes encoding O_2_^•−^ scavenging were ubiquitous, but the fractional gene allocation to O_2_^•−^ scavenging decreases as cell radius, and the co-varying total gene count increases, consistent with a nearly fixed set of core genes scavenging O_2_^•−^ that do not change with increasing gene count in larger cells (Hypothesis 1).

H_2_O_2_ has lower reactivity, longer intracellular and extracellular lifetimes and readily crosses cell membranes. Across eukaryotic phytoplankton, the fraction of the total genes encoding H_2_O_2_ producing and scavenging enzymes decreases with increasing cell radius (partially supports hypothesis 5). Presence of flagella and colony formation do not appear to influence H_2_O_2_ metabolism (contrary to hypotheses 3 & 4)

^•^NO has low reactivity, long intracellular and extracellular lifetimes and readily crosses cell membranes. Neither the fraction of the total genes for ^•^NO production nor for scavenging changed significantly with increasing cell radius, consistent with relatively low cytotoxicity and roles of ^•^NO in taxonomically diverse regulatory systems (contrary to hypothesis 5). Pennate diatoms frequently lack genes annotated as encoding ^•^NO producing enzymes, whereas centric diatoms frequently lack genes annotated as encoding ^•^NO scavenging enzymes (contrary to hypothesis 5). This finding is not explicable by differential diffusional losses of ^•^NO, but may reflect distinct roles of ^•^NO in the regulatory systems of diatom lineages.

## Supporting information

S1 Fig Violin plot presenting the range of log_10_ of the median cell radius in μm (‘log_Radius_um’) for each taxonomic lineage (‘Phylum’). Point colour corresponds to the source of the data, whether Genome or Transcriptome (‘Ome’). Violin width indicates the fraction of all datapoints occurring at a cell radius (‘log_Radius_um’) within a phylum. Citations for data sources are in S3 Table.
(TIF)

S2 Fig Longitude and latitude of isolation of analyzed organisms, overlaid on a world map Point colour corresponds to the taxonomic lineage (‘Phylum’). Ocean colour corresponds to depth Citations for data sources are in S3 Table. Data used to generate world map produced from the ‘ggOceanMaps’ R package [157].
(TIF)

S3 Fig Comparison of paired counts of particular genes encoding ROS production or scavenging from the genome (‘ROSGene_count.g’) or transcriptome (‘ROSGene_count.t’) taken from the same organism. Data was drawn from a subset of analyzed organisms for which both genome and transcriptome were available. Colour corresponds to the taxonomic lineage (‘Phylum’) Points are jittered to avoid overlapping, resulting in blocks around frequently occurring counts. Dashed line is at 1:1 where ‘ROSGene_count.g’ and ‘ROSGene_count.t’ would be equal. Citations for data sources are in S3 Table.
(TIF)

S4 Fig Comparison of paired counts of particular genes encoding ROS production or scavenging from manual and automatic annotations taken from the same organism. Data was drawn from a subset of genomes and transcriptomes which were both manually and automatically annotated. Colour corresponds to the ‘Gene’ Points are jittered to avoid overlapping, resulting in blocks around frequently occurring counts. Dashed line is placed at 1:1 where Manual and Automated counts would be equal. Citations for data sources in S4 Table.
(TIF)

S5 Fig Histogram of occurrences of number of total genes, in a genome or transcriptome, (y axis) that code for the production of enzymes that produce or scavenge H_2_O_2_, O2•− or ^•^NO in vivo. Symbol color corresponds to taxon lineage (‘Taxa’).
(TIF)

S6 Fig Comparison of log_10_ of the total number of ribosomal genes in an organism (‘log10(RibosomeCount)’) to log_10_ of the median cell radius in μm (‘log_Radius_um’). Colour corresponds to the taxonomic lineage (‘Phylum’), whereas symbol shape corresponds to the source of the data, whether Genome or Transcriptome (‘Ome’). Citations for data sources are in S3 Table.
(TIF)

S7 Fig Comparison of log_10_ (Total number of genes encoding H_2_O_2_, O2•− or ^•^NO metabolizing enzymes normalized to the total number of genes present in each Ochrophyte) vs. the log_10_(median cell radius in μm). Poisson (solid line) or Quasi-Poisson (dashed line) regressions fitted to data ± Standard Error (dotted line). Regressions were run without (blue line) ‘Colony’ and ‘Flagella’ as co-variates. Citations for data sources are in S3 Table.
(TIF)

S8 Fig Summary of O2•− scavenging enzymes encoded within genomes and transcriptomes of eukaryotic phytoplankton analyzedTaxa are ordered from top to bottom along the left according to increasing median cell diameter within each taxonomic lineage. Symbol colour corresponds to taxonomic lineages (‘Taxa’). Filled data points indicate that the data obtained from that organism was sourced from a genome, and unfilled data points were sourced from a transcriptome. The size of the symbol increases with the number of members of each enzyme found within each genome or transcriptome. Symbol absence means no sequences known to encode the enzyme family of interest were found in the target genome or transcriptome. The absence of transcripts encoding SOD from the Micromonas polaris transcriptome is likely due to low expression of SOD at the time that the mRNA was harvested for sequence analyses.
(TIF)

S9 Fig Summary of H_2_O_2_ metabolizing enzymes encoded within genomes of prokaryotic phytoplankton analyzed, faceted by whether the enzymes produce or scavenge H_2_O_2_. Taxa are ordered from top to bottom along the left according to increasing median cell diameter within each taxonomic lineage. Symbol colour corresponds to the genus of the prokaryote. Filled data points indicate that the data obtained from that organism was sourced from a genome. The size of the symbol increases with the number of members of each enzyme found within each genome or transcriptome. Symbol absence means no sequences known to encode the enzyme family of interest were found in the target genome or transcriptome.
(TIF)

S10 Fig Summary of H_2_O_2_ metabolizing enzymes encoded within genomes and transcriptomes of eukaryotic phytoplankton analyzed, faceted by whether the enzymes produce or scavenge H_2_O_2_. Taxa are ordered from top to bottom along the left according to increasing median cell diameter within each taxonomic lineage. Symbol colour corresponds to taxonomic lineages (‘Taxa’). Filled data points indicate that the data obtained from that organism was sourced from a genome, and unfilled data points were sourced from a transcriptome. The size of the symbol increases with the number of members of each enzyme found within each genome or transcriptome. Symbol absence means no sequences known to encode the enzyme family of interest were found in the target genome or transcriptome.
(TIF)

S11 Fig Summary of ^•^NO metabolizing enzymes encoded within genomes and transcriptomes of eukaryotic phytoplankton analyzed, faceted by whether the enzymes produce or scavenge ^•^NO. Taxa are ordered from top to bottom along the left according to increasing median cell diameter within each taxonomic lineage. Symbol colour corresponds to taxonomic lineages (‘Taxa’). Filled data points indicate that the data obtained from that organism was sourced from a genome, and unfilled data points were sourced from a transcriptome. The size of the symbol increases with the number of members of each enzyme found within each genome or transcriptome. Symbol absence means no sequences known to encode the enzyme family of interest were found in the target genome or transcriptome.
(TIF)

S12 Fig Comparison of the probability of having the genomic capacity to encode ^•^NO vs. the log_10_ (median cell radius in μm (‘log_Radius_um’)). Colony (solid line) or non-colony (dashed line) regressions fitted to data. Points along the y-axis indicate whether an organism has flagella (1) or does not have flagella (0).
(TIF)

S1 Table Variable names, definitions, units, and first location of occurrence in code, used for our data. (CSV)

S2 Table Enzyme commission number, kegg orthology number, enzyme name and ROS substrate metabolized. (CSV)

S3 Table Metadata for each organism. (CSV)

S4 Table Comparison of manual and automated gene counts. (CSV)

S1 File References for S1 to S4 Tables. (DOCX)
