# Supplementary figures and images for: Genomic capacities for Reactive Oxygen Species metabolism across marine phytoplankton
Source: PLoS One. 2023 Apr 25;18(4):e0284580. doi: 10.1371/journal.pone.0284580 (PMC10128935; doi:10.1371/journal.pone.0284580)

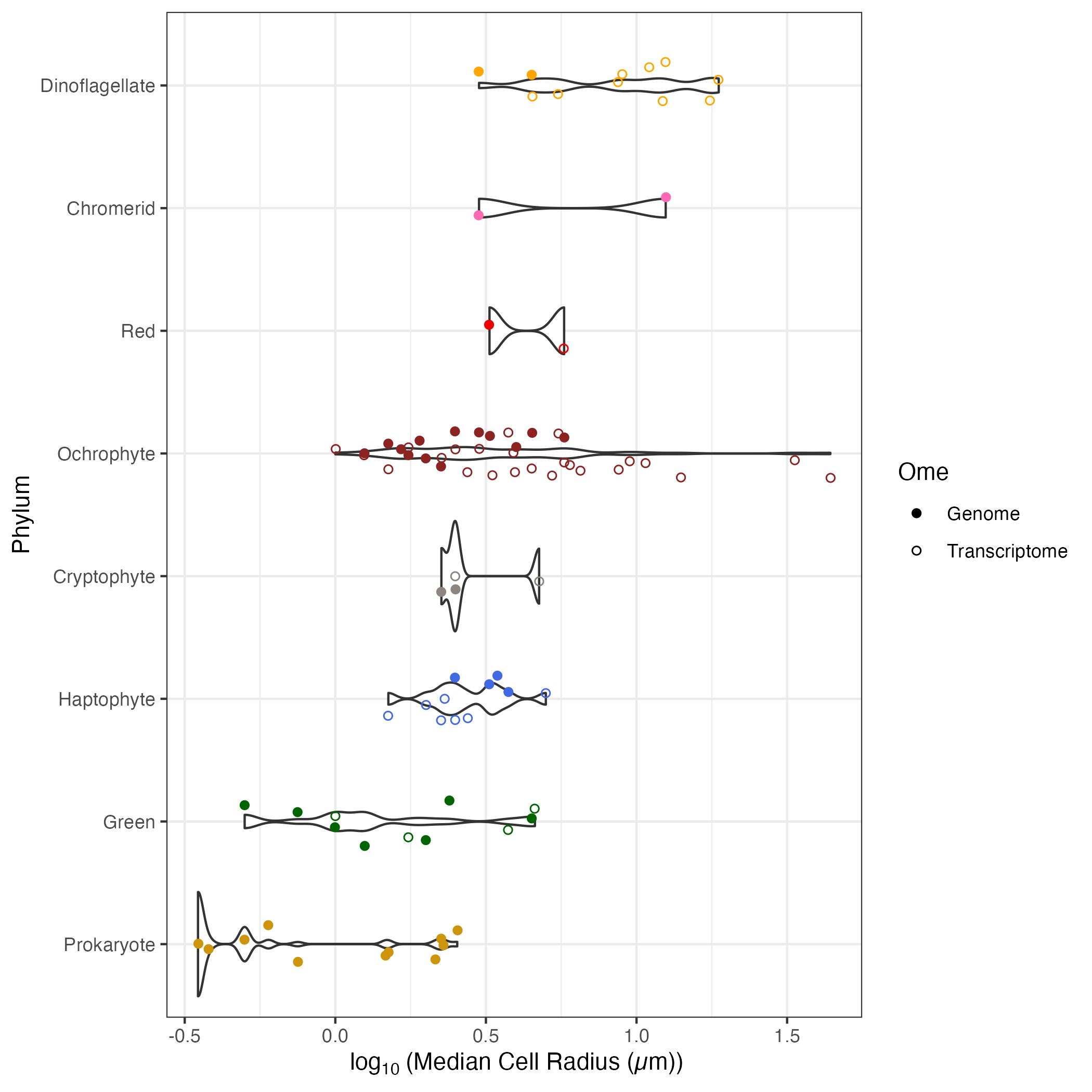

Supplement: S1 Fig — Point colour corresponds to the source of the data, whether Genome or Transcriptome (‘Ome’). Violin width indicates the fraction of all datapoints occurring at a cell radius (‘log_Radius_um’) within a phylum. Citations for data sources are in S3 Table. (TIF) [file pone.0284580.s001.tif]

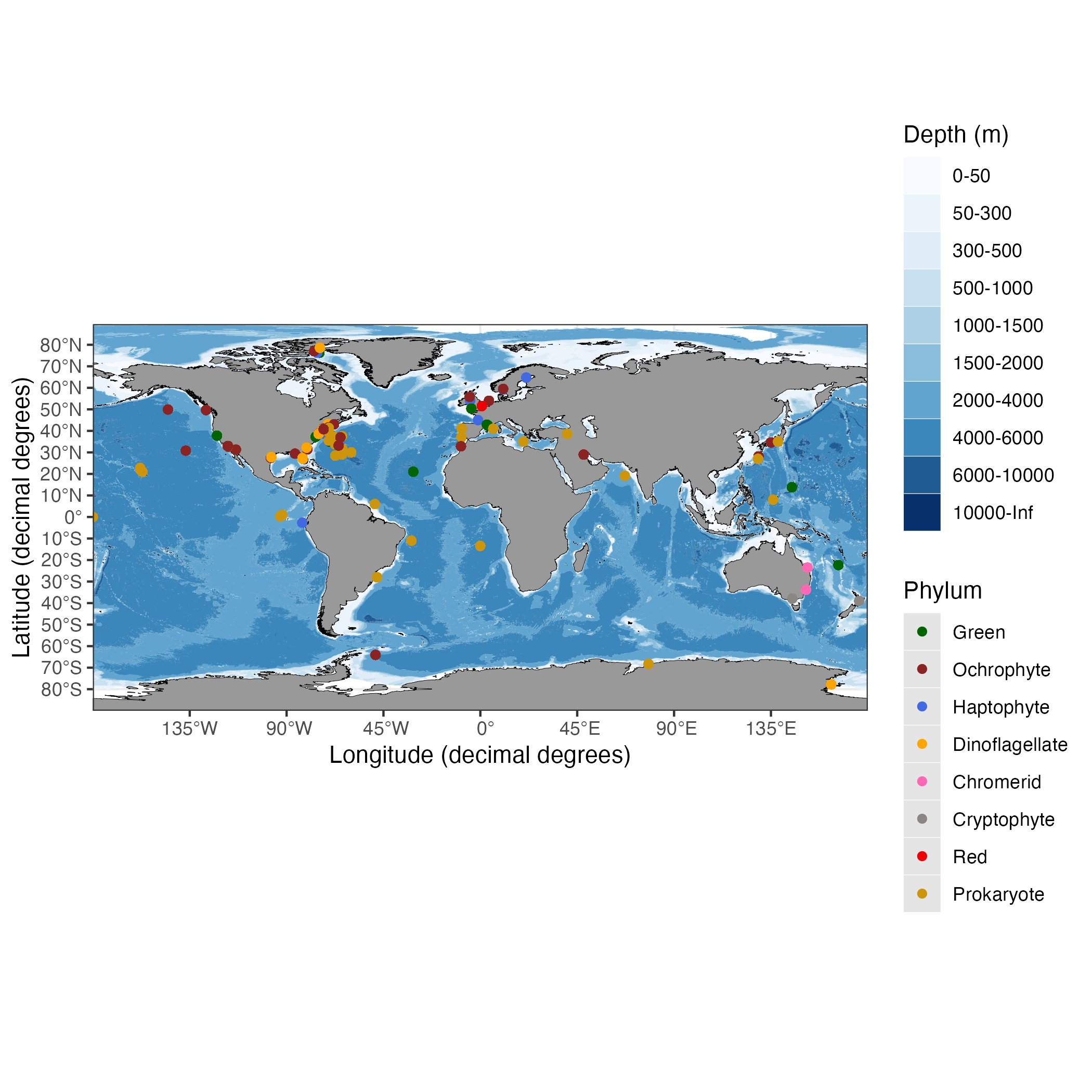

Supplement: S2 Fig — Ocean colour corresponds to depth Citations for data sources are in S3 Table. Data used to generate world map produced from the ‘ggOceanMaps’ R package [157]. (TIF) [file pone.0284580.s002.tif]

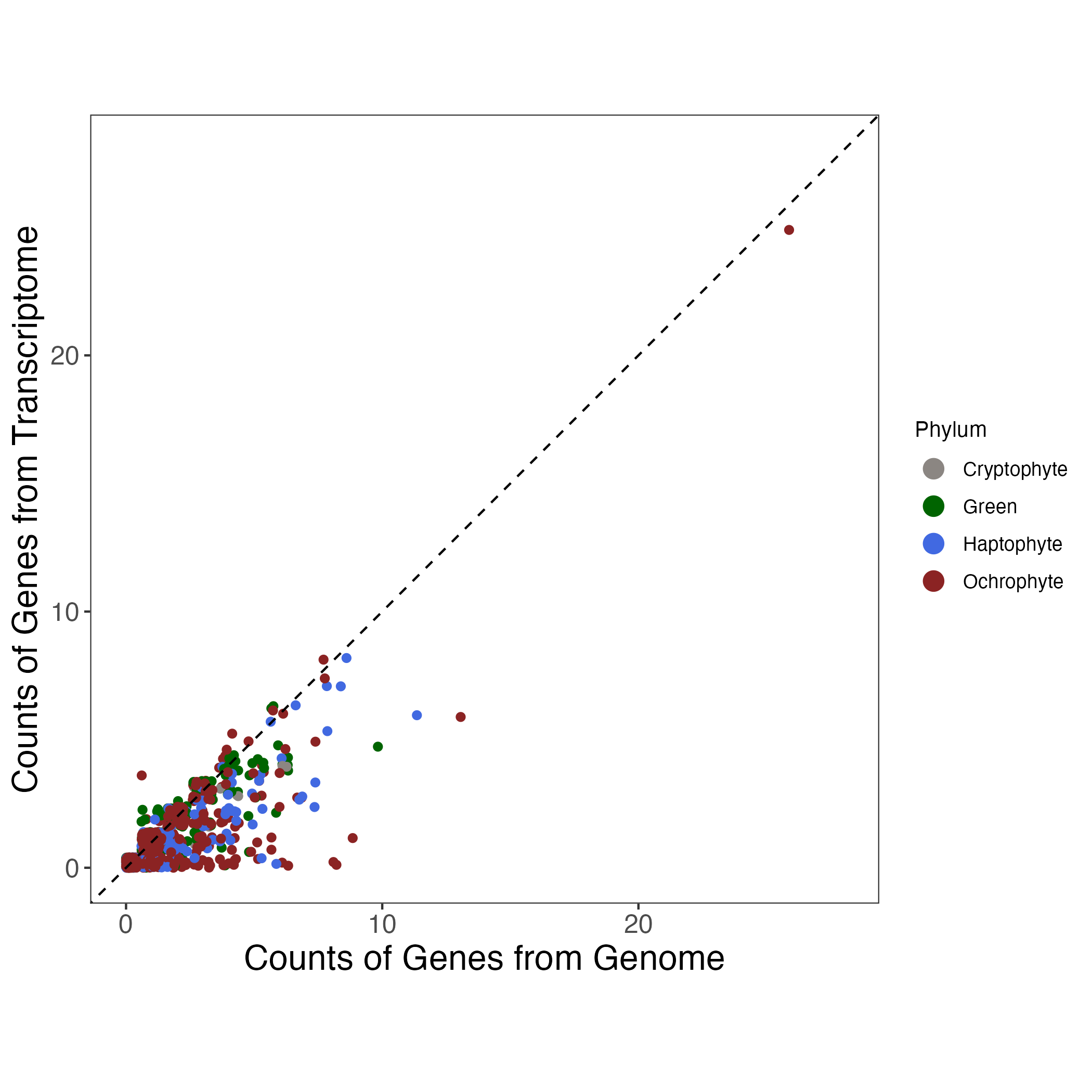

Supplement: S3 Fig — Data was drawn from a subset of analyzed organisms for which both genome and transcriptome were available. Colour corresponds to the taxonomic lineage (‘Phylum’) Points are jittered to avoid overlapping, resulting in blocks around frequently occurring counts. Dashed line is at 1:1 where ‘ROSGene_count.g’ and ‘ROSGene_count.t’ would be equal. Citations for data sources are in S3 Table. (TIF) [file pone.0284580.s003.tif]

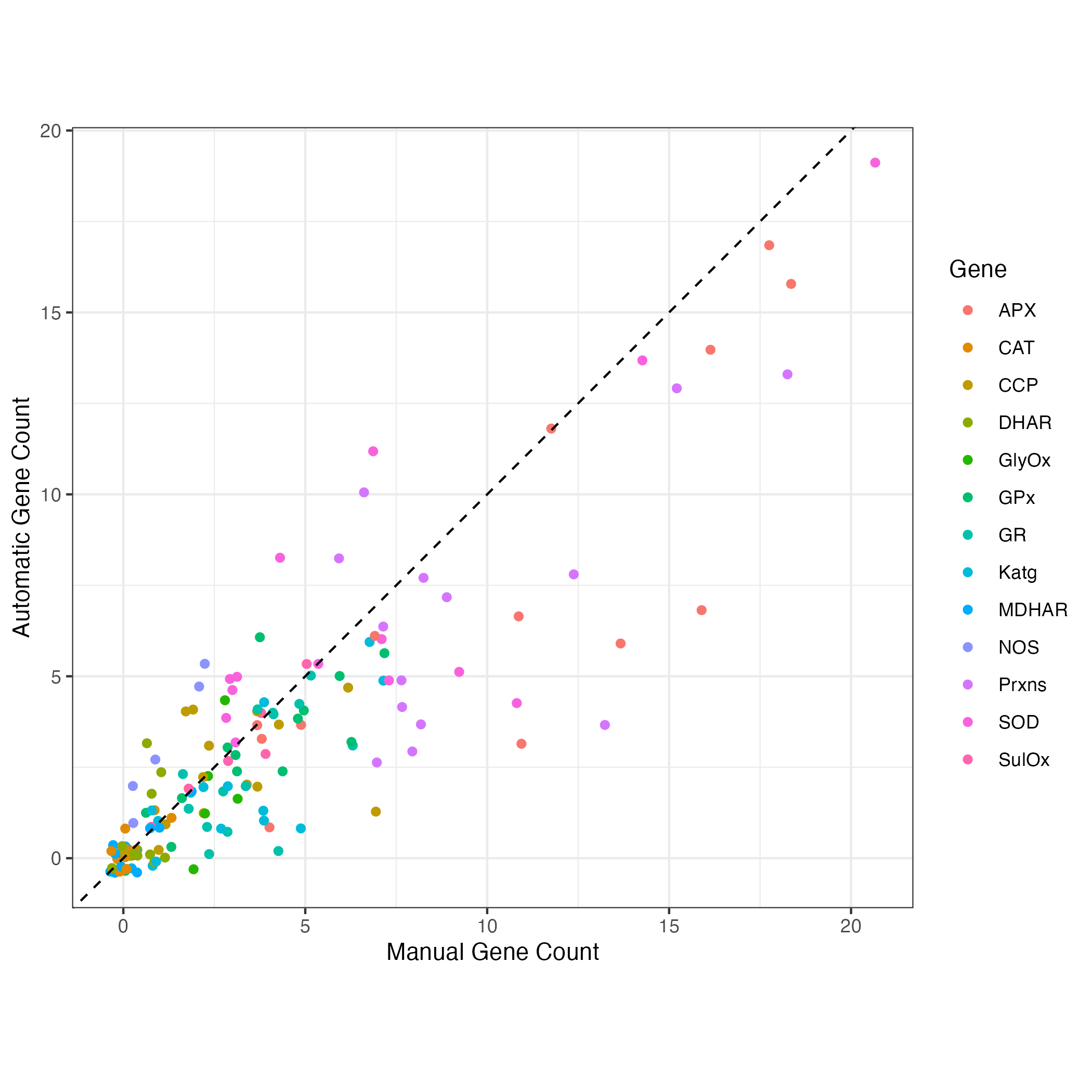

Supplement: S4 Fig — Data was drawn from a subset of genomes and transcriptomes which were both manually and automatically annotated. Colour corresponds to the ‘Gene’ Points are jittered to avoid overlapping, resulting in blocks around frequently occurring counts. Dashed line is placed at 1:1 where Manual and Automated counts would be equal. Citations for data sources in S4 Table. (TIF) [file pone.0284580.s004.tif]

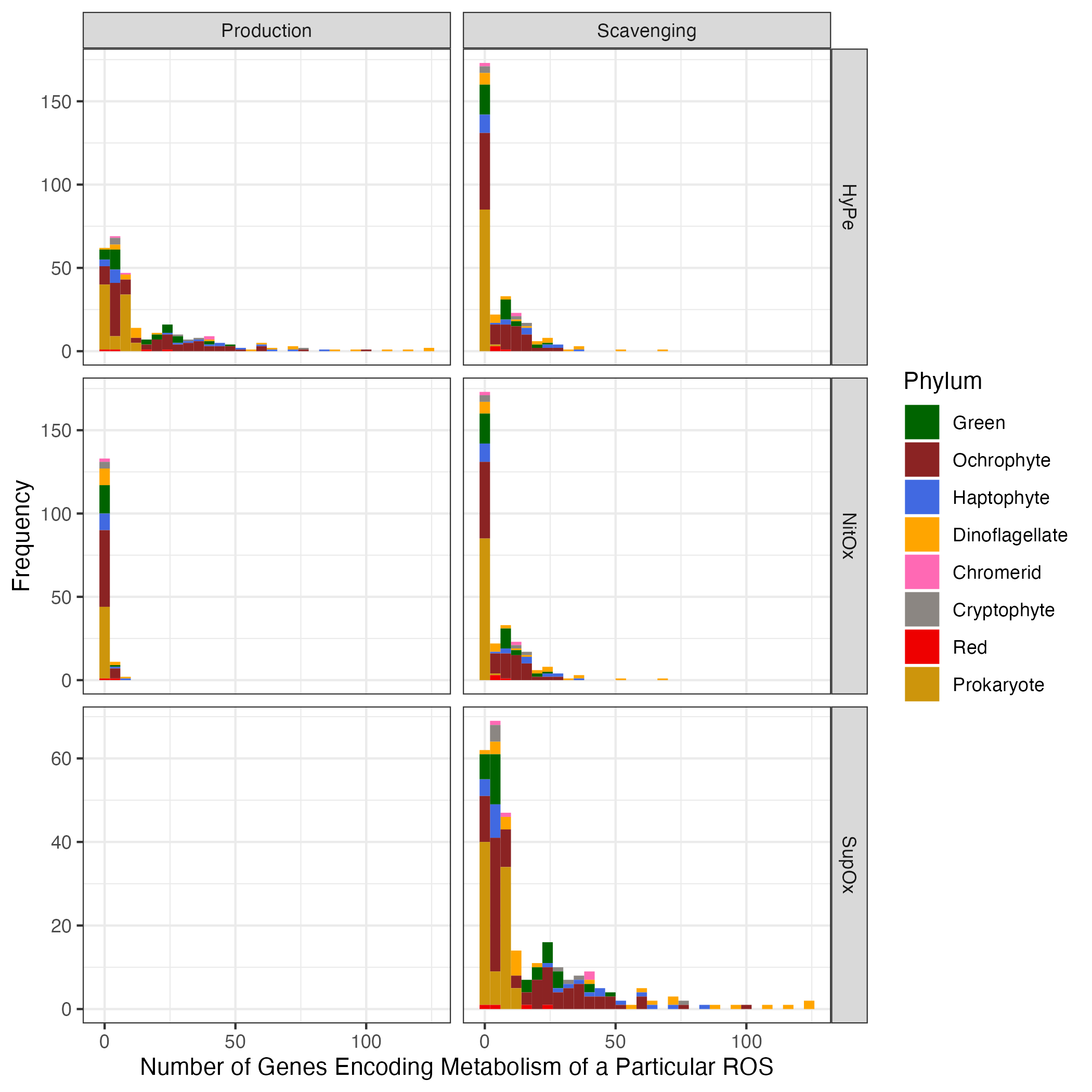

Supplement: S5 Fig — Symbol color corresponds to taxon lineage (‘Taxa’). (TIF) [file pone.0284580.s005.tif]

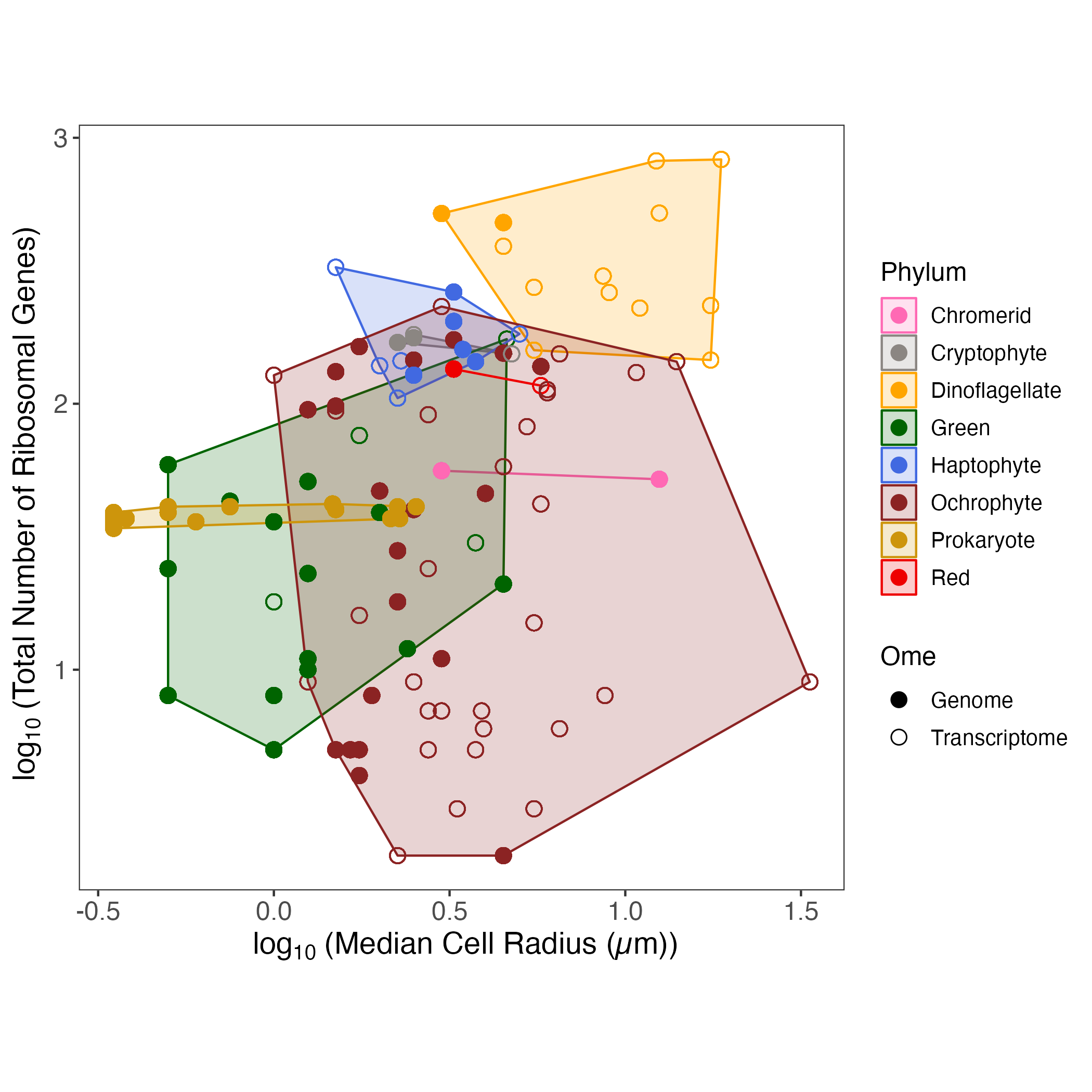

Supplement: S6 Fig — Colour corresponds to the taxonomic lineage (‘Phylum’), whereas symbol shape corresponds to the source of the data, whether Genome or Transcriptome (‘Ome’). Citations for data sources are in S3 Table. (TIF) [file pone.0284580.s006.tif]

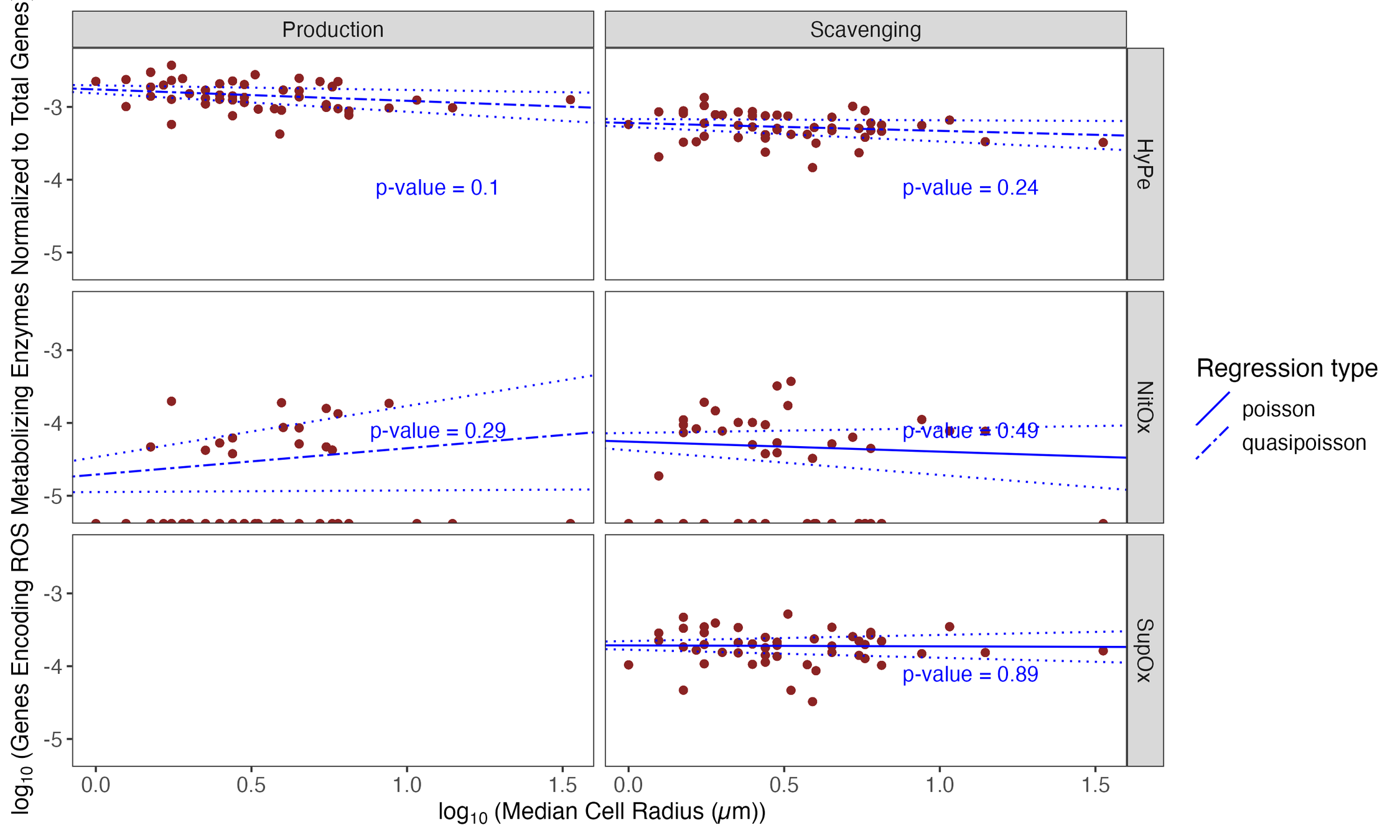

Supplement: S7 Fig — Poisson (solid line) or Quasi-Poisson (dashed line) regressions fitted to data ± Standard Error (dotted line). Regressions were run without (blue line) ‘Colony’ and ‘Flagella’ as co-variates. Citations for data sources are in S3 Table. (TIF) [file pone.0284580.s007.tif]

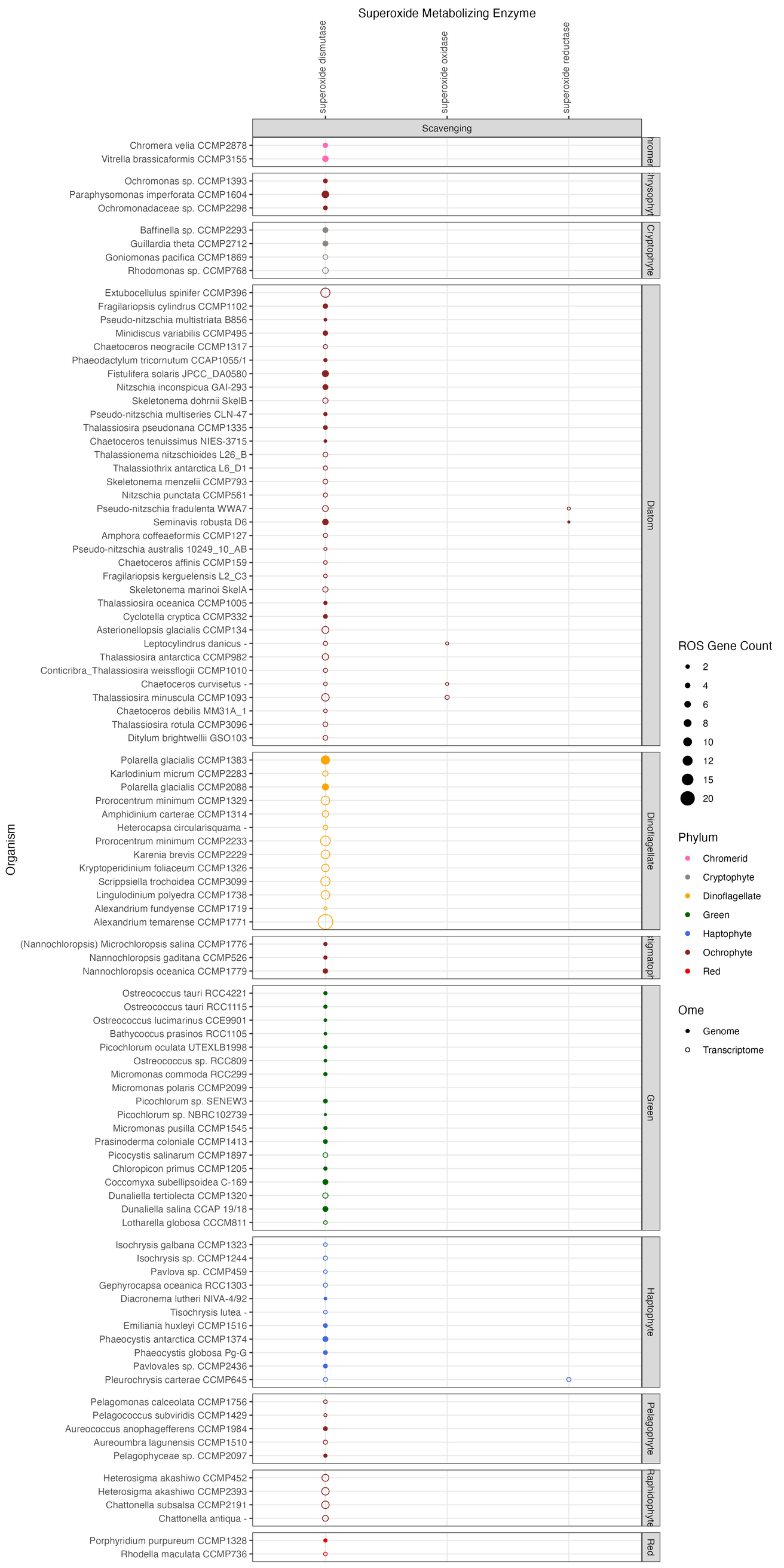

Supplement: S8 Fig — Symbol colour corresponds to taxonomic lineages (‘Taxa’). Filled data points indicate that the data obtained from that organism was sourced from a genome, and unfilled data points were sourced from a transcriptome. The size of the symbol increases with the number of members of each enzyme found within each genome or transcriptome. Symbol absence means no sequences known to encode the enzyme family of interest were found in the target genome or transcriptome. The absence of transcripts encoding SOD from the Micromonas polaris transcriptome is likely due to low expression of SOD at the time that the mRNA was harvested for sequence analyses. (TIF) [file pone.0284580.s008.tif]

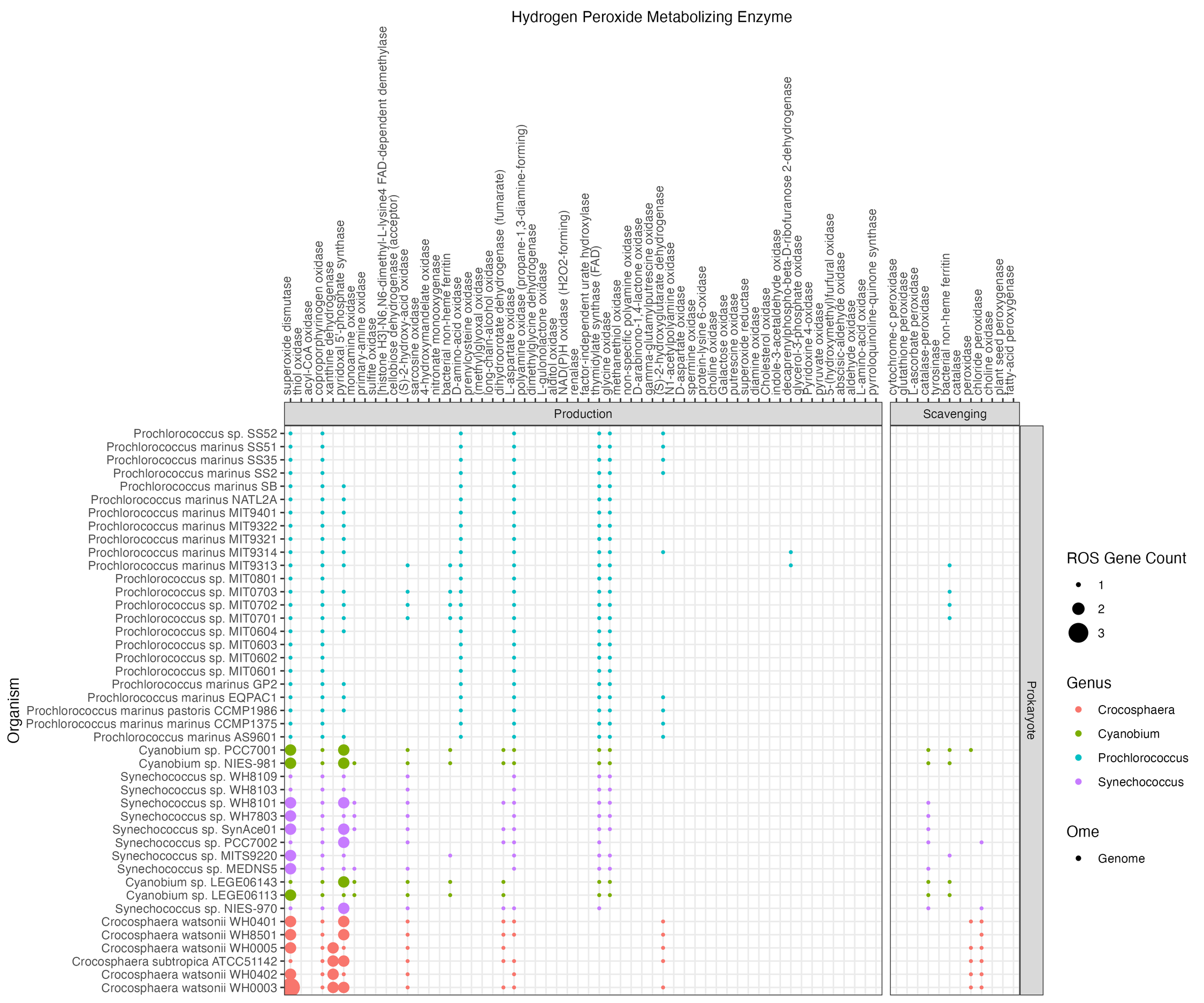

Supplement: S9 Fig — Taxa are ordered from top to bottom along the left according to increasing median cell diameter within each taxonomic lineage. Symbol colour corresponds to the genus of the prokaryote. Filled data points indicate that the data obtained from that organism was sourced from a genome. The size of the symbol increases with the number of members of each enzyme found within each genome or transcriptome. Symbol absence means no sequences known to encode the enzyme family of interest were found in the target genome or transcriptome. (TIF) [file pone.0284580.s009.tif]

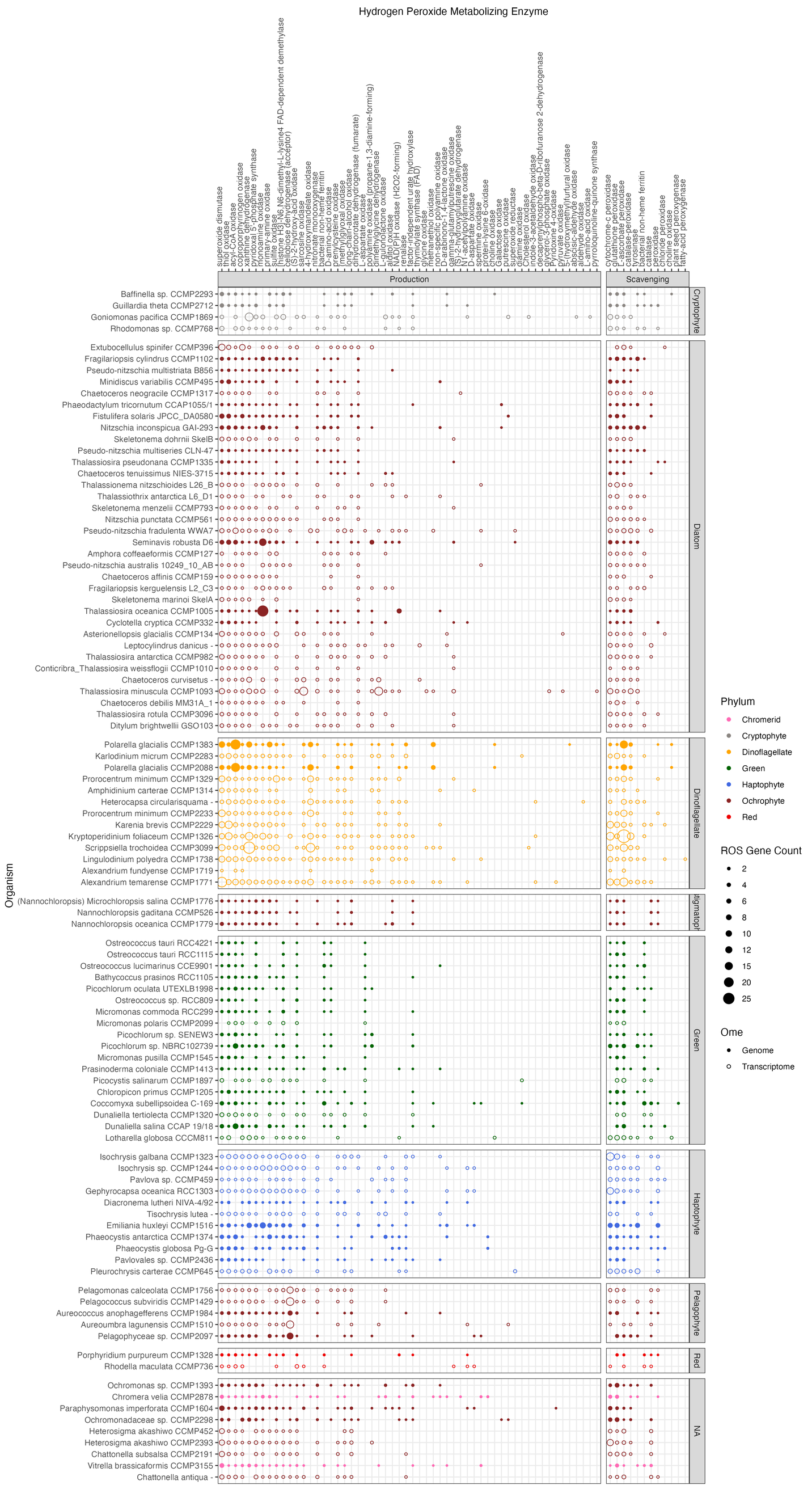

Supplement: S10 Fig — Taxa are ordered from top to bottom along the left according to increasing median cell diameter within each taxonomic lineage. Symbol colour corresponds to taxonomic lineages (‘Taxa’). Filled data points indicate that the data obtained from that organism was sourced from a genome, and unfilled data points were sourced from a transcriptome. The size of the symbol increases with the number of members of each enzyme found within each genome or transcriptome. Symbol absence means no sequences known to encode the enzyme family of interest were found in the target genome or transcriptome. (TIF) [file pone.0284580.s010.tif]

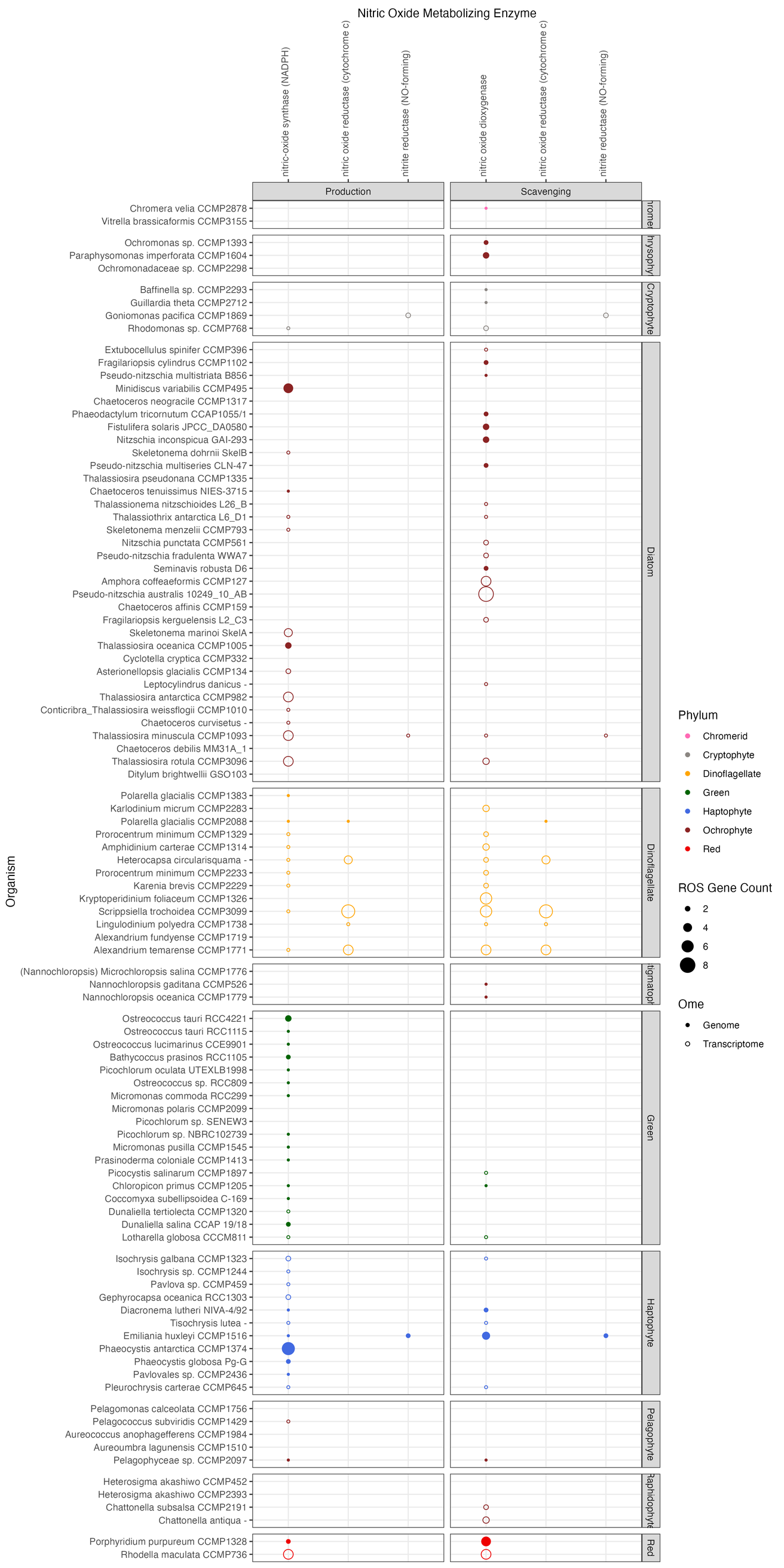

Supplement: S11 Fig — Taxa are ordered from top to bottom along the left according to increasing median cell diameter within each taxonomic lineage. Symbol colour corresponds to taxonomic lineages (‘Taxa’). Filled data points indicate that the data obtained from that organism was sourced from a genome, and unfilled data points were sourced from a transcriptome. The size of the symbol increases with the number of members of each enzyme found within each genome or transcriptome. Symbol absence means no sequences known to encode the enzyme family of interest were found in the target genome or transcriptome. (TIF) [file pone.0284580.s011.tif]

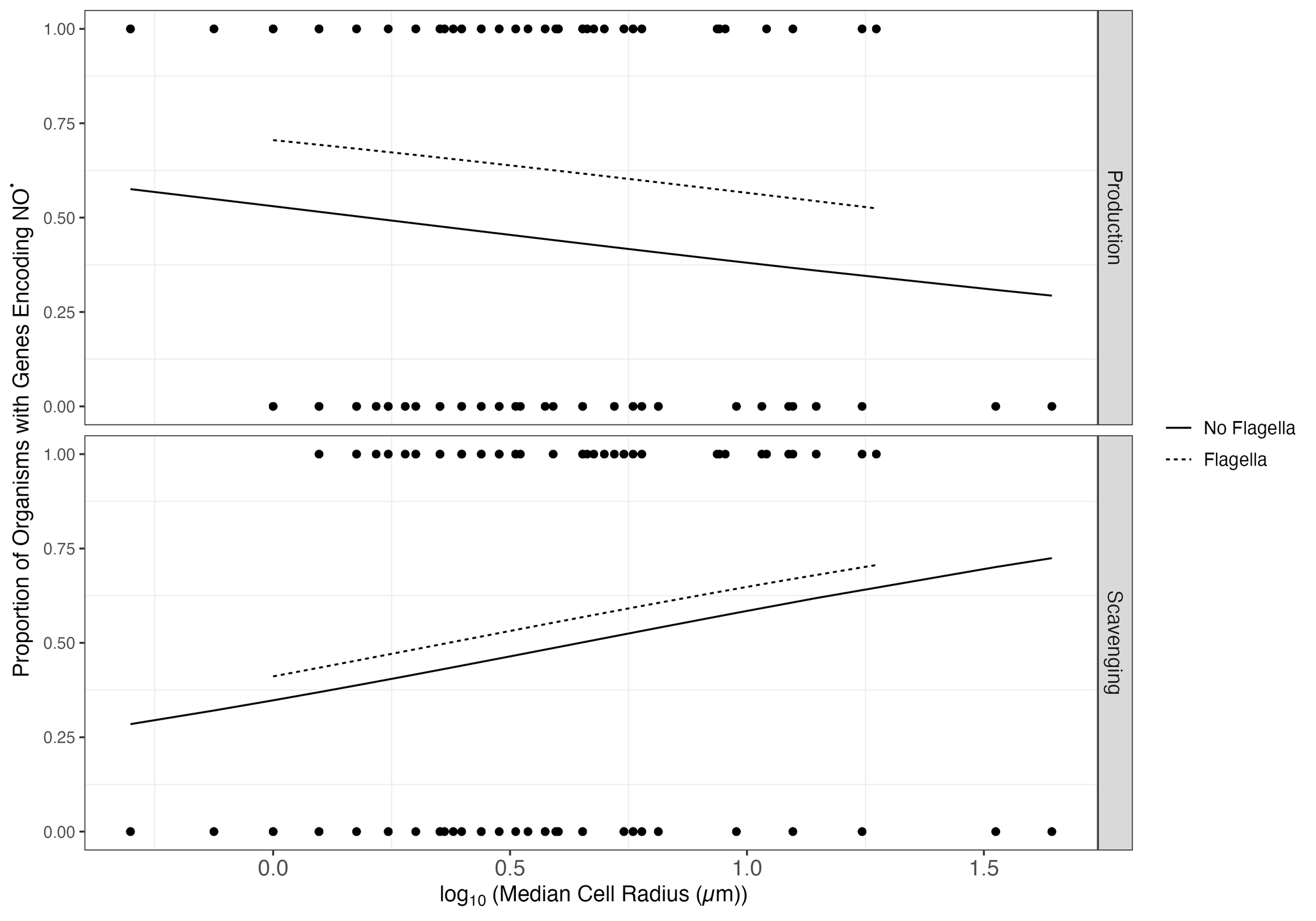

Supplement: S12 Fig — Colony (solid line) or non-colony (dashed line) regressions fitted to data. Points along the y-axis indicate whether an organism has flagella (1) or does not have flagella (0). (TIF) [file pone.0284580.s012.tif]
