# Supplementary material for: Genomic capacities for Reactive Oxygen Species metabolism across marine phytoplankton
Source: PLoS One. 2023 Apr 25;18(4):e0284580. doi: 10.1371/journal.pone.0284580 (PMC10128935; doi:10.1371/journal.pone.0284580)
Supplement: S1 File — (DOCX) [file pone.0284580.s017.docx]

Supplementary Table References

## References

1. Tao Z, Raffel RA, Souid A-K, Goodisman J. Kinetic Studies on Enzyme-Catalyzed Reactions: Oxidation of Glucose, Decomposition of Hydrogen Peroxide and Their Combination. Biophysical Journal. 2009;96: 2977–2988. doi:[10.1016/j.bpj.2008.11.071](https://doi.org/10.1016/j.bpj.2008.11.071)

2. Uwajima T, Shimizu Y, Terada O. Glycerol oxidase, a novel copper hemoprotein from *Aspergillus* *Japonicus.* Molecular and catalytic properties of the enzyme and its application to the analysis of serum triglycerides. J Biol Chem. 1984;259: 2748–2753. doi:[10.1016/S0021-9258(17)43209-1](https://doi.org/10.1016/S0021-9258(17)43209-1)

3. Hansen OC, Stougaard P. Hexose Oxidase from the Red Alga Chondrus crispus: PURIFICATION, MOLECULAR CLONING, AND EXPRESSION IN PICHIA PASTORIS*. J Biol Chem. 1997;272: 11581–11587. doi:[10.1074/jbc.272.17.11581](https://doi.org/10.1074/jbc.272.17.11581)

4. Pollegioni L, Piubelli L, Molla G. Cholesterol oxidase: Biotechnological applications: Biotechnological applications of sterol oxidase. FEBS Journal. 2009;276: 6857–6870. doi:[10.1111/j.1742-4658.2009.07379.x](https://doi.org/10.1111/j.1742-4658.2009.07379.x)

5. Serrano A, Carro J, Martínez AT. Reaction mechanisms and applications of aryl-alcohol oxidase. The Enzymes. Elsevier; 2020. pp. 167–192. doi:[10.1016/bs.enz.2020.05.005](https://doi.org/10.1016/bs.enz.2020.05.005)

6. Baron AJ, Stevens C, Wilmot C, Seneviratne KD, Blakeley V, Dooley DM, et al. Structure and mechanism of galactose oxidase. The free radical site. J Biol Chem. 1994;269: 25095–25105. doi:[10.1016/S0021-9258(17)31504-1](https://doi.org/10.1016/S0021-9258(17)31504-1)

7. Leitner C, Volc J, Haltrich D. Purification and Characterization of Pyranose Oxidase from the White Rot Fungus Trametes multicolor. Appl Environ Microbiol. 2001;67: 3636–3644. doi:[10.1128/AEM.67.8.3636-3644.2001](https://doi.org/10.1128/AEM.67.8.3636-3644.2001)

8. Schomburg D, Stephan D. L-Sorbose oxidase. In: Schomburg D, Stephan D, editors. Enzyme Handbook 10: Class 11: Oxidoreductases. Berlin, Heidelberg: Springer; 1995. pp. 397–399. doi:[10.1007/978-3-642-57756-7_107](https://doi.org/10.1007/978-3-642-57756-7_107)

9. Mugo AN, Kobayashi J, Yamasaki T, Mikami B, Ohnishi K, Yoshikane Y, et al. Crystal structure of pyridoxine 4-oxidase from *Mesorhizobium* *Loti*. Biochimica et Biophysica Acta (BBA) - Proteins and Proteomics. 2013;1834: 953–963. doi:[10.1016/j.bbapap.2013.03.004](https://doi.org/10.1016/j.bbapap.2013.03.004)

10. Hopkins TR, Muller F. Biochemistry of Alcohol Oxidase. In: van Verseveld HW, Duine JA, editors. Microbial Growth on C1 Compounds: Proceedings of the 5th International Symposium. Dordrecht: Springer Netherlands; 1987. pp. 150–157. doi:[10.1007/978-94-009-3539-6_19](https://doi.org/10.1007/978-94-009-3539-6_19)

11. Koolman J, Karlson P. Ecdysone Oxidase: Reaction and Specificity. European Journal of Biochemistry. 1978;89: 453–460. doi:[10.1111/j.1432-1033.1978.tb12548.x](https://doi.org/10.1111/j.1432-1033.1978.tb12548.x)

12. Pickl M, Fuchs M, Glueck SM, Faber K. The substrate tolerance of alcohol oxidases. Appl Microbiol Biotechnol. 2015;99: 6617–6642. doi:[10.1007/s00253-015-6699-6](https://doi.org/10.1007/s00253-015-6699-6)

13. Bhat SG, Vaidyanathan CS. Purification and Properties of l-4-Hydroxymandelate Oxidase from *Pseudomonas* *Convexa*. European Journal of Biochemistry. 1976;68: 323–331. doi:[10.1111/j.1432-1033.1976.tb10818.x](https://doi.org/10.1111/j.1432-1033.1976.tb10818.x)

14. Schomburg D, Stephan D. Long-chain-alcohol oxidase. In: Schomburg D, Stephan D, editors. Enzyme Handbook 10: Class 11: Oxidoreductases. Berlin, Heidelberg: Springer; 1995. pp. 438–443. doi:[10.1007/978-3-642-57756-7_116](https://doi.org/10.1007/978-3-642-57756-7_116)

15. Schomburg D, Stephan D. Hydroxyphytanate oxidase. In: Schomburg D, Stephan D, editors. Enzyme Handbook 10: Class 11: Oxidoreductases. Berlin, Heidelberg: Springer; 1995. pp. 468–470. doi:[10.1007/978-3-642-57756-7_123](https://doi.org/10.1007/978-3-642-57756-7_123)

16. Lobo-da-Cunha A, Amaral-de-Carvalho D, Oliveira E, Alves Â, Costa V, Calado G. Mannitol oxidase and polyol dehydrogenases in the digestive gland of gastropods: Correlations with phylogeny and diet. PLOS ONE. 2018;13: e0193078. doi:[10.1371/journal.pone.0193078](https://doi.org/10.1371/journal.pone.0193078)

17. Rothe GM. Aldehyde oxidase isoenzymes (E.C. 1.2.3.1) in potato tubers (*Solanum* *Tuberosum*). Plant and Cell Physiology. 1974. doi:[10.1093/oxfordjournals.pcp.a075029](https://doi.org/10.1093/oxfordjournals.pcp.a075029)

18. PUNDIR CS, VERMA U. Isolation, purification, immobilization of oxalate oxidase and its clinical applications. Hindustan antibiotics bulletin. 1993;35.

19. Choy WH. The gut microbiome and metabolic pathways of recurrent kidney stone patients and their non-stone-forming live-in partners. 2018. doi:[10.14288/1.0371254](https://doi.org/10.14288/1.0371254)

20. Rahman AYA, Usharraj AO, Misra BB, Thottathil GP, Jayasekaran K, Feng Y, et al. Draft genome sequence of the rubber tree *Hevea* *Brasiliensis*. BMC Genomics. 2013;14: 75. doi:[10.1186/1471-2164-14-75](https://doi.org/10.1186/1471-2164-14-75)

21. Silaghi-Dumitrescu R, Ng KY, Viswanathan R, Kurtz DM. A Flavo-Diiron Protein from Desulfovibrio vulgaris with Oxidase and Nitric Oxide Reductase Activities. Evidence for an in Vivo Nitric Oxide Scavenging Function. Biochemistry. 2005;44: 3572–3579. doi:[10.1021/bi0477337](https://doi.org/10.1021/bi0477337)

22. Blomberg MRA, Siegbahn PEM. Improved free energy profile for reduction of NO in cytochrome c dependent nitric oxide reductase (cNOR). J Comput Chem. 2016;37: 1810–1818. doi:[10.1002/jcc.24396](https://doi.org/10.1002/jcc.24396)

23. Re3data.Org. Bigelow National Center for Algae and Microbiota. 2017. doi:[10.17616/R3PN76](https://doi.org/10.17616/R3PN76)

24. Hill DRA, Wetherbee R. *Guillardia Theta* gen. Et sp.nov. (Cryptophyceae). Can J Bot. 1990;68: 1873–1876. doi:[10.1139/b90-245](https://doi.org/10.1139/b90-245)

25. Curtis BA, Tanifuji G, Burki F, Gruber A, Irimia M, Maruyama S, et al. Algal genomes reveal evolutionary mosaicism and the fate of nucleomorphs. Nature. 2012;492: 59–65. doi:[10.1038/nature11681](https://doi.org/10.1038/nature11681)

26. Karlson B, Andreasson A, Johansen M, Karlberg M, Loo A, Skjevik A-T. Nordic Microalgae. http://nordicmicroalgae.org; 2020.

27. Iwasa K, Shimizu A. Motility of the diatom, *Phaeodactylum* *Tricornutum*. Experimental Cell Research. 1972;74: 552–558. doi:[10.1016/0014-4827(72)90416-8](https://doi.org/10.1016/0014-4827(72)90416-8)

28. Bowler C, Allen AE, Badger JH, Grimwood J, Jabbari K, Kuo A, et al. The *Phaeodactylum* genome reveals the evolutionary history of diatom genomes. Nature. 2008;456: 239–244. doi:[10.1038/nature07410](https://doi.org/10.1038/nature07410)

29. Ova Ozcan D, Ovez B. Evaluation of the interaction of temperature and light intensity on the growth of *Phaeodactylum* *Tricornutum*: Kinetic modeling and optimization. Biochemical Engineering Journal. 2020;154: 107456. doi:[10.1016/j.bej.2019.107456](https://doi.org/10.1016/j.bej.2019.107456)

30. Matsumoto M, Mayama S, Nemoto M, Fukuda Y, Muto M, Yoshino T, et al. Morphological and molecular phylogenetic analysis of the high triglyceride-producing marine diatom, *Fistulifera* *Solaris Sp. Nov. (Bacillariophyceae)*. Phycological Research. 2014;62: 257–268. doi:[10.1111/pre.12066](https://doi.org/10.1111/pre.12066)

31. Nanjappa D, Sanges R, Ferrante MI, Zingone A. Diatom flagellar genes and their expression during sexual reproduction in *Leptocylindrus* *Danicus*. BMC Genomics. 2017;18: 813. doi:[10.1186/s12864-017-4210-8](https://doi.org/10.1186/s12864-017-4210-8)

32. Tanaka T, Maeda Y, Veluchamy A, Tanaka M, Abida H, Maréchal E, et al. Oil accumulation by the oleaginous diatom *Fistulifera* *Solaris* as revealed by the genome and transcriptome. Plant Cell. 2015;27: 162–176. doi:[10.1105/tpc.114.135194](https://doi.org/10.1105/tpc.114.135194)

33. Misumi O, Yoshida Y, Nishida K, Fujiwara T, Sakajiri T, Hirooka S, et al. Genome analysis and its significance in four unicellular algae, *Cyanidioshyzon* *Merolae*, *Ostreococcus* *Tauri*, *Chlamydomonas* *Reinhardtii*, and *Thalassiosira* *Pseudonana*. Journal of Plant Research. 2008;121: 3–17. doi:[10.1007/s10265-007-0133-9](https://doi.org/10.1007/s10265-007-0133-9)

34. Armbrust EV, Berges JA, Bowler C, Green BR, Martinez D, Putnam NH, et al. The Genome of the Diatom *Thalassiosira Pseudonana*: Ecology, Evolution, and Metabolism. Science. 2004;306: 79–86. doi:[10.1126/science.1101156](https://doi.org/10.1126/science.1101156)

35. Hevia-Orube J, Orive E, David H, Díez A, Laza-Martínez A, Miguel I, et al. Molecular and morphological analyses of solitary forms of brackish Thalassiosiroid diatoms (Coscinodiscophyceae), with emphasis on their phenotypic plasticity. European Journal of Phycology. 2016;51: 11–30. doi:[10.1080/09670262.2015.1077394](https://doi.org/10.1080/09670262.2015.1077394)

36. Lommer M, Roy A-S, Schilhabel M, Schreiber S, Rosenstiel P, LaRoche J. Recent Transfer of an Iron-Regulated Gene from the Plastid to the Nuclear Genome in an Oceanic Diatom Adapted to Chronic Iron Limitation. BMC Genomics. 2010;11: 718. doi:[10.1186/1471-2164-11-718](https://doi.org/10.1186/1471-2164-11-718)

37. Bucciarelli E, Pondaven P, Sarthou G. Effects of an iron-light co-limitation on the elemental composition (Si, C, N) of the marine diatoms &lt;i&gt;Thalassiosira oceanica&lt;/i&gt; and &lt;i&gt;Ditylum brightwellii&lt;/i&gt; Biogeochemistry: Open Ocean; 2009 Jul. doi:[10.5194/bgd-6-7175-2009](https://doi.org/10.5194/bgd-6-7175-2009)

38. Johnson LK, Alexander H, Brown CT. Re-assembly, quality evaluation, and annotation of 678 microbial eukaryotic reference transcriptomes. GigaScience. 2019;8. doi:[10.1093/gigascience/giy158](https://doi.org/10.1093/gigascience/giy158)

39. Miklasz KA. Physical Constraints on the Size and Shape of Microalgae. PhD thesis, Stanford University. 2012.

40. Phyto’pedia. Phyto’pedia - The Phytoplankton Encyclopedia Project. https://www.eoas.ubc.ca/research/phytoplankton/index.html; 2012.

41. Keeling PJ, Burki F, Wilcox HM, Allam B, Allen EE, Amaral-Zettler LA, et al. The Marine Microbial Eukaryote Transcriptome Sequencing Project (MMETSP): Illuminating the Functional Diversity of Eukaryotic Life in the Oceans through Transcriptome Sequencing. PLOS Biology. 2014;12: e1001889. doi:[10.1371/journal.pbio.1001889](https://doi.org/10.1371/journal.pbio.1001889)

42. Gaonkar CC, Piredda R, Sarno D, Zingone A, Montresor M, Kooistra WHCF. Species detection and delineation in the marine planktonic diatoms Chaetoceros and Bacteriastrum through metabarcoding: Making biological sense of haplotype diversity. Environmental Microbiology. 2020;22: 1917–1929. doi:[10.1111/1462-2920.14984](https://doi.org/10.1111/1462-2920.14984)

43. Traller JC, Cokus SJ, Lopez DA, Gaidarenko O, Smith SR, McCrow JP, et al. Genome and methylome of the oleaginous diatom *Cyclotella* *Cryptica* reveal genetic flexibility toward a high lipid phenotype. Biotechnology for Biofuels. 2016;9: 258. doi:[10.1186/s13068-016-0670-3](https://doi.org/10.1186/s13068-016-0670-3)

44. Guo L, Liang S, Zhang Z, Liu H, Wang S, Pan K, et al. Genome assembly of Nannochloropsis oceanica provides evidence of host nucleus overthrow by the symbiont nucleus during speciation. Communications Biology. 2019;2: 1–12. doi:[10.1038/s42003-019-0500-9](https://doi.org/10.1038/s42003-019-0500-9)

45. Vieler A, Wu G, Tsai C-H, Bullard B, Cornish AJ, Harvey C, et al. Genome, Functional Gene Annotation, and Nuclear Transformation of the Heterokont Oleaginous Alga Nannochloropsis oceanica CCMP1779. PLOS Genetics. 2012;8: e1003064. doi:[10.1371/journal.pgen.1003064](https://doi.org/10.1371/journal.pgen.1003064)

46. Suda S, Atsumi M, Miyashita H. Taxonomic characterization of a marine *Nannochloropsis* species, *N. Oceanica Sp.* Nov. (Eustigmatophyceae). Phycologia. 2002;41: 273–279. doi:[10.2216/i0031-8884-41-3-273.1](https://doi.org/10.2216/i0031-8884-41-3-273.1)

47. Radakovits R, Jinkerson RE, Fuerstenberg SI, Tae H, Settlage RE, Boore JL, et al. Draft genome sequence and genetic transformation of the oleaginous alga *Nannochloropis* *Gaditana*. Nat Commun. 2012;3: 686. doi:[10.1038/ncomms1688](https://doi.org/10.1038/ncomms1688)

48. Mitra M, Patidar SK, George B, Shah F, Mishra S. A euryhaline *Nannochloropsis* *Gaditana* with potential for nutraceutical (EPA) and biodiesel production. Algal Research. 2015;8: 161–167. doi:[10.1016/j.algal.2015.02.006](https://doi.org/10.1016/j.algal.2015.02.006)

49. Ohan JA, Hovde BT, Zhang XL, Davenport KW, Chertkov O, Han C, et al. Nuclear Genome Assembly of the Microalga *Nannochloropsis* *Salina* CCMP1776. Microbiology Resource Announcements. 2019;8: e00750–19. doi:[10.1128/MRA.00750-19](https://doi.org/10.1128/MRA.00750-19)

50. Oliver A, Podell S, Pinowska A, Traller JC, Smith SR, McClure R, et al. Diploid genomic architecture of *Nitzschia* *Inconspicua*, an elite biomass production diatom. Sci Rep. 2021;11: 15592. doi:[10.1038/s41598-021-95106-3](https://doi.org/10.1038/s41598-021-95106-3)

51. Krienitz L, Krienitz D, Dadheech PK, Hübener T, Kotut K, Luo W, et al. Food algae for Lesser Flamingos: A stocktaking. Hydrobiologia. 2016;775: 21–50. doi:[10.1007/s10750-016-2706-x](https://doi.org/10.1007/s10750-016-2706-x)

52. Vaulot D, Gall F, Le Marie D, Guillou L, Partensky F. The Roscoff Culture Collection (RCC): A collection dedicated to marine picoplankton. nova_hedwigia. 2004;79: 49–70. doi:[10.1127/0029-5035/2004/0079-0049](https://doi.org/10.1127/0029-5035/2004/0079-0049)

53. Palenik B, Grimwood J, Aerts A, Rouzé P, Salamov A, Putnam N, et al. The tiny eukaryote *Ostreococcus* provides genomic insights into the paradox of plankton speciation. PNAS. 2007;104: 7705–7710. doi:[10.1073/pnas.0611046104](https://doi.org/10.1073/pnas.0611046104)

54. Blanc-Mathieu R, Krasovec M, Hebrard M, Yau S, Desgranges E, Martin J, et al. Population genomics of picophytoplankton unveils novel chromosome hypervariability. Sci Adv. 2017;3: e1700239. doi:[10.1126/sciadv.1700239](https://doi.org/10.1126/sciadv.1700239)

55. Ooijen G van, Knox K, Kis K, Bouget F-Y, Millar AJ. Genomic Transformation of the Picoeukaryote Ostreococcus tauri. JoVE (Journal of Visualized Experiments). 2012; e4074. doi:[10.3791/4074](https://doi.org/10.3791/4074)

56. Moreau H, Verhelst B, Couloux A, Derelle E, Rombauts S, Grimsley N, et al. Gene functionalities and genome structure in *Bathycoccus* *Prasinos* reflect cellular specializations at the base of the green lineage. Genome Biology. 2012;13: R74. doi:[10.1186/gb-2012-13-8-r74](https://doi.org/10.1186/gb-2012-13-8-r74)

57. Marin B, Melkonian M. Molecular Phylogeny and Classification of the Mamiellophyceae class. Nov. (Chlorophyta) based on Sequence Comparisons of the Nuclear- and Plastid-encoded rRNA Operons. Protist. 2010;161: 304–336. doi:[10.1016/j.protis.2009.10.002](https://doi.org/10.1016/j.protis.2009.10.002)

58. Throndsen J. The Planktonic Marine Flagellates. Identifying Marine Phytoplankton. Elsevier; 1997. pp. 591–729. doi:[10.1016/B978-012693018-4/50007-0](https://doi.org/10.1016/B978-012693018-4/50007-0)

59. Worden AZ, Lee J-H, Mock T, Rouzé P, Simmons MP, Aerts AL, et al. Green evolution and dynamic adaptations revealed by genomes of the marine picoeukaryotes *Micromonas*. Science. 2009;324: 268–272. doi:[10.1126/science.1167222](https://doi.org/10.1126/science.1167222)

60. Šlapeta J, López-García P, Moreira D. Global Dispersal and Ancient Cryptic Species in the Smallest Marine Eukaryotes. Mol Biol Evol. 2006;23: 23–29. doi:[10.1093/molbev/msj001](https://doi.org/10.1093/molbev/msj001)

61. Blanc G, Agarkova I, Grimwood J, Kuo A, Brueggeman A, Dunigan DD, et al. The genome of the polar eukaryotic microalga *Coccomyxa* *Subellipsoidea* reveals traits of cold adaptation. Genome Biol. 2012;13: R39. doi:[10.1186/gb-2012-13-5-r39](https://doi.org/10.1186/gb-2012-13-5-r39)

62. Takahashi K, Ide Y, Hayakawa J, Yoshimitsu Y, Fukuhara I, Abe J, et al. Lipid productivity in TALEN-induced starchless mutants of the unicellular green alga *Coccomyxa* *Sp.* Strain Obi. Algal Research. 2018;32: 300–307. doi:[10.1016/j.algal.2018.04.020](https://doi.org/10.1016/j.algal.2018.04.020)

63. Heimann K, Huerlimann R. Chapter 3 - Microalgal Classification: Major Classes and Genera of Commercial Microalgal Species. In: Kim S-K, editor. Handbook of Marine Microalgae. Boston: Academic Press; 2015. pp. 25–41. doi:[10.1016/B978-0-12-800776-1.00003-0](https://doi.org/10.1016/B978-0-12-800776-1.00003-0)

64. Oren A. A hundred years of *Dunaliella* research: 1905. Saline Systems. 2005;1: 2. doi:[10.1186/1746-1448-1-2](https://doi.org/10.1186/1746-1448-1-2)

65. Polle JEW, Barry K, Cushman J, Schmutz J, Tran D, Hathwaik LT, et al. Draft Nuclear Genome Sequence of the Halophilic and Beta-Carotene-Accumulating Green Alga *Dunaliella* *Salina* Strain CCAP19/18. Genome Announc. 2017;5. doi:[10.1128/genomeA.01105-17](https://doi.org/10.1128/genomeA.01105-17)

66. dos Santos AL, Pollina T, Gourvil P, Corre E, Marie D, Garrido JL, et al. Chloropicophyceae, a New Class of Picophytoplanktonic Prasinophytes. Scientific Reports. 2017;7: 1–20. doi:[10.1038/s41598-017-12412-5](https://doi.org/10.1038/s41598-017-12412-5)

67. Krienitz L, Bock C, Kotut K, Luo W. *Picocystis Salinarum* (Chlorophyta) in saline lakes and hot springs of East Africa. Phycologia. 2012;51: 22–32. doi:[10.2216/11-28.1](https://doi.org/10.2216/11-28.1)

68. Borowitzka MA. Biology of Microalgae. Microalgae in Health and Disease Prevention. Elsevier; 2018. pp. 23–72. doi:[10.1016/B978-0-12-811405-6.00003-7](https://doi.org/10.1016/B978-0-12-811405-6.00003-7)

69. Read BA, Kegel J, Klute MJ, Kuo A, Lefebvre SC, Maumus F, et al. Pan genome of the phytoplankton *Emiliania* underpins its global distribution. Nature. 2013;499: 209–213. doi:[10.1038/nature12221](https://doi.org/10.1038/nature12221)

70. Sekino K, Kobayashi H, Shiraiwa Y. Role of Coccoliths in the Utilization of Inorganic Carbon by a Marine Unicellular Coccolithophorid, *Emiliania* *Huxleyi*: A Survey Using Intact Cells and Protoplasts. Plant Cell Physiol. 1996;37: 123–127. doi:[10.1093/oxfordjournals.pcp.a028921](https://doi.org/10.1093/oxfordjournals.pcp.a028921)

71. Bendif EM, Probert I, Schroeder DC, de Vargas C. On the Description of *Tisochrysis* *Lutea* Gen. Nov. Sp. Nov. And *Isochrysis* *Nuda* Sp. Nov. In the Isochrysidales, and the Transfer of *Dicrateria* to the Prymnesiales (Haptophyta). J Appl Phycol. 2013;25: 1763–1776. doi:[10.1007/s10811-013-0037-0](https://doi.org/10.1007/s10811-013-0037-0)

72. Carrier G, Baroukh C, Rouxel C, Duboscq-Bidot L, Schreiber N, Bougaran G. Draft genomes of the algae Tisochrysis lutea strains. SEANOE; 2017. doi:[10.17882/47171](https://doi.org/10.17882/47171)

73. Beltrami E. Chapter 9 - Viral Outbreaks and Blood Clots. In: Beltrami E, editor. Mathematical Models for Society and Biology (Second Edition). Boston: Academic Press; 2013. pp. 159–186. doi:[10.1016/B978-0-12-404624-5.00009-1](https://doi.org/10.1016/B978-0-12-404624-5.00009-1)

74. Caron DA, Dennett MR, Moran DM, Schaffner RA, Lonsdale DJ, Gobler CJ, et al. Development and Application of a Monoclonal-Antibody Technique for Counting Aureococcus anophagefferens, an Alga Causing Recurrent Brown Tides in the Mid-Atlantic United States. Appl Environ Microbiol. 2003;69: 5492–5502. doi:[10.1128/AEM.69.9.5492-5502.2003](https://doi.org/10.1128/AEM.69.9.5492-5502.2003)

75. Gobler CJ, Berry DL, Dyhrman ST, Wilhelm SW, Salamov A, Lobanov AV, et al. Niche of harmful alga Aureococcus anophagefferens revealed through ecogenomics. Proc Natl Acad Sci U S A. 2011;108: 4352–4357. doi:[10.1073/pnas.1016106108](https://doi.org/10.1073/pnas.1016106108)

76. Sieburth JMcN, Johnson PW, Hargraves PE. Ultrastructure and Ecology of *Aureococcus* *Anophageferens* Gen. Et Sp. Nov. (Chrysophyceae): The Dominant Picoplankter During a Bloom in Narragansett Bay, Rhode Island, Summer 19851. J Phycol. 1988;24: 416–425. doi:[10.1111/j.1529-8817.1988.tb04485.x](https://doi.org/10.1111/j.1529-8817.1988.tb04485.x)

77. Ribeiro CG, Santos AL dos, Gourvil P, Gall FL, Marie D, Tragin M, et al. Culturable diversity of Arctic phytoplankton during pack ice melting. bioRxiv. 2019; 642264. doi:[10.1101/642264](https://doi.org/10.1101/642264)

78. Roy S, editor. Phytoplankton pigments: Characterization, chemotaxonomy, and applications in oceanography. Cambridge ; New York: Cambridge University Press; 2011.

79. Bhattacharya D, Price DC, Chan CX, Qiu H, Rose N, Ball S, et al. Genome of the red alga *Porphyridium* *Purpureum*. Nat Commun. 2013;4: 1941. doi:[10.1038/ncomms2931](https://doi.org/10.1038/ncomms2931)

80. Markina ZV, Orlova TYu, Vasyanovich YA, Vardavas AI, Stivaktakis PD, Vardavas CI, et al. Porphyridium purpureum microalga physiological and ultrastructural changes under copper intoxication. Toxicology Reports. 2021;8: 988–993. doi:[10.1016/j.toxrep.2021.04.015](https://doi.org/10.1016/j.toxrep.2021.04.015)

81. Partensky F, Hess WR, Vaulot D. *Prochlorococcus*, a Marine Photosynthetic Prokaryote of Global Significance. Microbiol Mol Biol Rev. 1999;63: 106–127.

82. Aguilo-Ferretjans M del M, Bosch R, Puxty RJ, Latva M, Zadjelovic V, Chhun A, et al. Pili allow dominant marine cyanobacteria to avoid sinking and evade predation. Nat Commun. 2021;12: 1857. doi:[10.1038/s41467-021-22152-w](https://doi.org/10.1038/s41467-021-22152-w)

83. Kettler GC, Martiny AC, Huang K, Zucker J, Coleman ML, Rodrigue S, et al. Patterns and implications of gene gain and loss in the evolution of *Prochlorococcus*. PLoS Genet. 2007;3: e231. doi:[10.1371/journal.pgen.0030231](https://doi.org/10.1371/journal.pgen.0030231)

84. Park JS, Han J, Suh S-S, Kim H-J, Lee T-K, Jung SW. Characterization of bacterial community structure in two alcyonacean soft corals (*Litophyton* *Sp.* And *Sinularia* *Sp.*) From Chuuk, Micronesia. Coral Reefs. 2021. doi:[10.1007/s00338-021-02176-w](https://doi.org/10.1007/s00338-021-02176-w)

85. Dufresne A, Salanoubat M, Partensky F, Artiguenave F, Axmann IM, Barbe V, et al. Genome sequence of the cyanobacterium Prochlorococcus marinus SS120, a nearly minimal oxyphototrophic genome. Proc Natl Acad Sci USA. 2003;100: 10020–10025. doi:[10.1073/pnas.1733211100](https://doi.org/10.1073/pnas.1733211100)

86. Rocap G, Larimer FW, Lamerdin J, Malfatti S, Chain P, Ahlgren NA, et al. Genome divergence in two *Prochlorococcus* ecotypes reflects oceanic niche differentiation. Nature. 2003;424: 1042–1047. doi:[10.1038/nature01947](https://doi.org/10.1038/nature01947)

87. Gu H, Zeng N, Xie Z, Wang D, Wang W, Yang W. Morphology, phylogeny, and toxicity of Atama complex (Dinophyceae) from the Chukchi Sea. Polar Biol. 2013;36: 427–436. doi:[10.1007/s00300-012-1273-5](https://doi.org/10.1007/s00300-012-1273-5)

88. Lim AS, Jeong HJ, Ok JH, Kim SJ. Feeding by the harmful phototrophic dinoflagellate *Takayama* *Tasmanica* (Family Kareniaceae). Harmful Algae. 2018;74: 19–29. doi:[10.1016/j.hal.2018.03.009](https://doi.org/10.1016/j.hal.2018.03.009)

89. Sharma VK, Rhudy KB, Millero FJ. Diurnal variation of texas “brown tide” (*Aureoumbra* *Lagunensis*) in relation to metals. Journal of Environmental Science and Health, Part A. 2000;35: 1077–1088. doi:[10.1080/10934520009377021](https://doi.org/10.1080/10934520009377021)

90. Harvey EL, Menden-Deuer S, Rynearson TA. Persistent Intra-Specific Variation in Genetic and Behavioral Traits in the Raphidophyte, Heterosigma akashiwo. Front Microbiol. 2015;6.

91. Dursun F, Taş S, Koray T. Spring bloom of the raphidophycean Heterosigma akashiwo in the Golden Horn Estuary at the northeast of Sea of Marmara. EgeJFAS. 2016;33: 201. doi:[10.12714/egejfas.2016.33.3.03](https://doi.org/10.12714/egejfas.2016.33.3.03)

92. Wang C, Lan CQ. Effects of shear stress on microalgae A review. Biotechnology Advances. 2018;36: 986–1002. doi:[10.1016/j.biotechadv.2018.03.001](https://doi.org/10.1016/j.biotechadv.2018.03.001)

93. Iba W. Isolation and Growth of Dinoglagellate, *Scrippsiella* *Sp.* And diatom, *Melosira* *Cf. Moniliformis* in controlled conditions. Indones Aquac J. 2014;9: 55–63. doi:[10.15578/iaj.9.1.2014.55-63](https://doi.org/10.15578/iaj.9.1.2014.55-63)

94. Kawachi M. Microbial Culture Collection, National Institute for Environmental Studies. National Institute of Genetics, ROIS; 2021. doi:[10.15468/8RML10](https://doi.org/10.15468/8RML10)

95. Yamaguchi H, Shimura Y, Suzuki S, Yamagishi T, Tatarazako N, Kawachi M. Complete Genome Sequence of *Cyanobium* *Sp.* NIES-981, a Marine Strain Potentially Useful for Ecotoxicological Bioassays. Genome Announc. 2016;4: e00736–16. doi:[10.1128/genomeA.00736-16](https://doi.org/10.1128/genomeA.00736-16)

96. Albrecht M, Pröschold T, Schumann R. Identification of Cyanobacteria in a Eutrophic Coastal Lagoon on the Southern Baltic Coast. Front Microbiol. 2017;8: 923. doi:[10.3389/fmicb.2017.00923](https://doi.org/10.3389/fmicb.2017.00923)

97. Hirakawa Y, Howe A, James ER, Keeling PJ. Morphological Diversity between Culture Strains of a Chlorarachniophyte, *Lotharella* *Globosa*. PLOS ONE. 2011;6: e23193. doi:[10.1371/journal.pone.0023193](https://doi.org/10.1371/journal.pone.0023193)

98. Ota S, Vaulot D, Gall FL, Yabuki A, Ishida K. *Partenskyella Glossopodia* gen. Et sp. Nov., The First Report of a Chlorarachniophyte that Lacks a Pyrenoid. Protist. 2009;160: 137–150. doi:[10.1016/j.protis.2008.09.003](https://doi.org/10.1016/j.protis.2008.09.003)

99. Biller SJ, Berube PM, Berta-Thompson JW, Kelly L, Roggensack SE, Awad L, et al. Genomes of diverse isolates of the marine cyanobacterium *Prochlorococcus*. Sci Data. 2014;1: 140034. doi:[10.1038/sdata.2014.34](https://doi.org/10.1038/sdata.2014.34)

100. Shimada A, Nishijima M, Maruyama T. Seasonal appearance of *Prochlorococcus* in Suruga Bay, Japan in 1992. J Oceanogr. 1995;51: 289–300. doi:[10.1007/BF02285167](https://doi.org/10.1007/BF02285167)

101. Urbach E, Scanlan DJ, Distel DL, Waterbury JB, Chisholm SW. Rapid Diversification of Marine Picophytoplankton with Dissimilar Light-Harvesting Structures Inferred from Sequences of *Prochlorococcus* and *Synechococcus* (Cyanobacteria). J Mol Evol. 1998;46: 188–201. doi:[10.1007/PL00006294](https://doi.org/10.1007/PL00006294)

102. Rocap G, Distel DL, Waterbury JB, Chisholm SW. Resolution of *Prochlorococcus* and *Synechococcus* Ecotypes by Using 16S-23S Ribosomal DNA Internal Transcribed Spacer Sequences. Appl Environ Microbiol. 2002;68: 1180–1191. doi:[10.1128/AEM.68.3.1180-1191.2002](https://doi.org/10.1128/AEM.68.3.1180-1191.2002)

103. Brahamsha B. An abundant cell-surface polypeptide is required for swimming by the nonflagellated marine cyanobacterium *Synechococcus*. Proc Natl Acad Sci U S A. 1996;93: 6504–6509.

104. Rosales N, Ortega J, Mora R, Morales E. Influence of salinity on the growth and biochemical composition of the cyanobacterium *Synechococcus* *Sp.* Ciencias Marinas. 2005;31: 349–355. doi:[10.7773/cm.v31i2.59](https://doi.org/10.7773/cm.v31i2.59)

105. Wei Y, Sun J, Zhang X, Wang J, Huang K. Picophytoplankton size and biomass around equatorial eastern Indian Ocean. Microbiologyopen. 2018;8: e00629. doi:[10.1002/mbo3.629](https://doi.org/10.1002/mbo3.629)

106. Marston MF, Polson SW. Whole-Genome Sequence of the Cyanobacterium *Synechococcus* sp. Strain WH 8101. Stewart FJ, editor. Microbiol Resour Announc. 2020;9. doi:[10.1128/MRA.01593-19](https://doi.org/10.1128/MRA.01593-19)

107. Shimura Y, Hirose Y, Misawa N, Wakazuki S, Fujisawa T, Nakamura Y, et al. Complete Genome Sequence of a Coastal Cyanobacterium, *Synechococcus* *Sp.* Strain NIES-970. Genome Announc. 2017;5: e00139–17. doi:[10.1128/genomeA.00139-17](https://doi.org/10.1128/genomeA.00139-17)

108. Ramos V, Morais J, Castelo-Branco R, Pinheiro Â, Martins J, Regueiras A, et al. Cyanobacterial diversity held in microbial biological resource centers as a biotechnological asset: The case study of the newly established LEGE culture collection. J Appl Phycol. 2018;30: 1437–1451. doi:[10.1007/s10811-017-1369-y](https://doi.org/10.1007/s10811-017-1369-y)

109. Wilde A, Mullineaux CW. Motility in cyanobacteria: Polysaccharide tracks and Type IV pilus motors. Molecular Microbiology. 2015;98: 998–1001. doi:[10.1111/mmi.13242](https://doi.org/10.1111/mmi.13242)

110. Vogt RA, Ignoffo TR, Sullivan LJ, Herndon J, Stillman JH, Kimmerer WJ. Feeding capabilities and limitations in the nauplii of two pelagic estuarine copepods, *Pseudodiaptomus* *Marinus* and *Oithona* *Davisae*. Limnol Oceanogr. 2013;58: 2145–2157. doi:[10.4319/lo.2013.58.6.2145](https://doi.org/10.4319/lo.2013.58.6.2145)

111. Mock T, Otillar RP, Strauss J, McMullan M, Paajanen P, Schmutz J, et al. Evolutionary genomics of the cold-adapted diatom *Fragilariopsis* *Cylindrus*. Nature. 2017;541: 536–540. doi:[10.1038/nature20803](https://doi.org/10.1038/nature20803)

112. Findlay CR, Wiens R, Rak M, Sedlmair J, J. Hirschmugl C, Morrison J, et al. Rapid biodiagnostic ex vivo imaging at 1 $M$m pixel resolution with thermal source FTIR FPA. Analyst. 2015;140: 2493–2503. doi:[10.1039/C4AN01982B](https://doi.org/10.1039/C4AN01982B)

113. D’Alelio D, Amato A, Luedeking A, Montresor M. Sexual and vegetative phases in the planktonic diatom Pseudo-nitzschia multistriata. Harmful Algae. 2009;8: 225–232. doi:[10.1016/j.hal.2008.05.004](https://doi.org/10.1016/j.hal.2008.05.004)

114. Ferrante IM. Pseudo-nitzschia multistriata strain B856, whole genome shotgun sequencing project. NCBI Direct Sumbission. 2019.

115. Orlova TYu, Stonik IV, Aizdaicher NA, Bates SS, Léger C, Fehling J. Toxicity, morphology and distribution of *Pseudo-nitzschia* *Calliantha*, *P. Multistriata* and *P. Multiseries* (Bacillariophyta) from the northwestern Sea of Japan. Botanica Marina. 2008;51. doi:[10.1515/BOT.2008.035](https://doi.org/10.1515/BOT.2008.035)

116. Davidovich NA, Bates SS. Patterns of Sexual Reproduction in the Pennate Diatoms Pseudo-nitzschia multiseries AND P. pseudodelicatissima. 1998; 4.

117. Lundholm N, Daugbjerg N, Moestrup Ø. Phylogeny of the Bacillariaceae with emphasis on the genus *Pseudo* - *Nitzschia* (Bacillariophyceae) based on partial LSU rDNA. European Journal of Phycology. 2002;37: 115–134. doi:[10.1017/S096702620100347X](https://doi.org/10.1017/S096702620100347X)

118. Kociolek P. Nitzschia palea. In Diatoms of North America. https://diatoms.org/species/nitzschia_palea; 2011.

119. Garacci M, Barret M, Folgoas C, Flahaut E, Chimowa G, Bertucci A, et al. Transcriptomic response of the benthic freshwater diatom *Nitzschia* *Palea* exposed to Few Layer Graphene. Environmental Science: Nano. 2019;6: 1363–1381. doi:[10.1039/C8EN00987B](https://doi.org/10.1039/C8EN00987B)

120. Crowell RM, Nienow JA, Cahoon AB. The complete chloroplast and mitochondrial genomes of the diatom *Nitzschia* *Palea* $<\setminus$*i*$>$ *(Bacillariophyceae) Demonstrate High Sequence Similarity to the Endosymbiont Organelles of the Dinotom* *Durinskia* *Baltica* $<\setminus$*i*$>$. J Phycol. 2019;55: 352–364. doi:[10.1111/jpy.12824](https://doi.org/10.1111/jpy.12824)

121. Kessenich CR, Ruck EC, Schurko AM, Wickett NJ, Alverson AJ. Transcriptomic Insights into the Life History of Bolidophytes, the Sister Lineage to Diatoms. J Phycol. 2014;50: 977–983. doi:[10.1111/jpy.12222](https://doi.org/10.1111/jpy.12222)

122. Mahadik GA, Castellani C, Mazzocchi MG. Effect of diatom morphology on the small-scale behavior of the copepod Temora stylifera (Dana, 1849). J Exp Mar Biol Ecol. 2017;493: 41–48. doi:[10.1016/j.jembe.2017.05.001](https://doi.org/10.1016/j.jembe.2017.05.001)

123. Gérikas Ribeiro C, dos Santos AL, Gourvil P, Le Gall F, Marie D, Tragin M, et al. Culturable diversity of Arctic phytoplankton during pack ice melting. Deming JW, Michel C, editors. Elementa: Science of the Anthropocene. 2020;8. doi:[10.1525/elementa.401](https://doi.org/10.1525/elementa.401)

124. Balzano S, Percopo I, Siano R, Gourvil P, Chanoine M, Marie D, et al. Morphological and genetic diversity of Beaufort Sea diatoms with high contributions from the *Chaetoceros* *Neogracilis* species complex. J Phycol. 2017;53: 161–187. doi:[10.1111/jpy.12489](https://doi.org/10.1111/jpy.12489)

125. Fernandes LF, Frassão-Santos EK. Mucilaginous species of *Thalassiosira* $<\setminus$*i*$>$ *Cleve* *Emend: Hasle (Diatomeae) in* *South Brazilian* *Waters*. Acta Bot Bras. 2011;25: 31–42. doi:[10.1590/S0102-33062011000100006](https://doi.org/10.1590/S0102-33062011000100006)

126. McFarland M, Nayak AR, Stockley N, Twardowski M, Sullivan J. Enhanced Light Absorption by Horizontally Oriented Diatom Colonies. Front Mar Sci. 2020;7: 494. doi:[10.3389/fmars.2020.00494](https://doi.org/10.3389/fmars.2020.00494)

127. Finenko ZZ, Krupatkina-Akinina DK. Effect of inorganic phosphorus on the growth rate of diatoms. Mar Biol. 1974;26: 193–201. doi:[10.1007/BF00389251](https://doi.org/10.1007/BF00389251)

128. Lajeunesse TC, Parkinson JE, Reimer JD. A Genetics-Based Description of *Symbiodinium* *Minutum* Sp. Nov. And *S. Psygmophilum* Sp. Nov. (Dinophyceae), Two Dinoflagellates Symbiotic with Cnidaria. J Phycol. 2012;48: 1380–1391. doi:[10.1111/j.1529-8817.2012.01217.x](https://doi.org/10.1111/j.1529-8817.2012.01217.x)

129. Shinzato C, Mungpakdee S, Satoh N, Shoguchi E. A genomic approach to coral-dinoflagellate symbiosis: Studies of *Acropora* *Digitifera* and *Symbiodinium* *Minutum*. Front Microbiol. 2014;5. doi:[10.3389/fmicb.2014.00336](https://doi.org/10.3389/fmicb.2014.00336)

130. Shoguchi E, Shinzato C, Kawashima T, Gyoja F, Mungpakdee S, Koyanagi R, et al. Draft Assembly of the *Symbiodinium* *Minutum* Nuclear Genome Reveals Dinoflagellate Gene Structure. Current Biology. 2013;23: 1399–1408. doi:[10.1016/j.cub.2013.05.062](https://doi.org/10.1016/j.cub.2013.05.062)

131. Kirk AL, Clowez S, Lin F, Grossman AR, Xiang T. Transcriptome Reprogramming of Symbiodiniaceae *Breviolum* *Minutum* in Response to Casein Amino Acids Supplementation. Front Physiol. 2020;11: 574654. doi:[10.3389/fphys.2020.574654](https://doi.org/10.3389/fphys.2020.574654)

132. Chen W-NU, Hsiao Y-J, Mayfield AB, Young R, Hsu L-L, Peng S-E. Transmission of a heterologous clade C Symbiodinium in a model anemone infection system via asexual reproduction. PeerJ. 2016;4. doi:[10.7717/peerj.2358](https://doi.org/10.7717/peerj.2358)

133. Martinez S, Kolodny Y, Shemesh E, Scucchia F, Nevo R, Levin-Zaidman S, et al. Energy Sources of the Depth-Generalist Mixotrophic Coral *StylophoraPistillata* . Front Mar Sci. 2020;7: 988. doi:[10.3389/fmars.2020.566663](https://doi.org/10.3389/fmars.2020.566663)

134. Montresor M, Lovejoy C, Orsini L, Procaccini G, Roy S. Bipolar distribution of the cyst-forming dinoflagellate *Polarella* *Glacialis*. Polar Biol. 2003;26: 186–194. doi:[10.1007/s00300-002-0473-9](https://doi.org/10.1007/s00300-002-0473-9)

135. Stephens TG, González-Pech RA, Cheng Y, Mohamed AR, Bhattacharya D, Ragan MA, et al. *Polarella Glacialis* genomes encode tandem repeats of single-exon genes with functions critical to adaptation of dinoflagellates. bioRxiv. 2019; 704437. doi:[10.1101/704437](https://doi.org/10.1101/704437)

136. Thomson PG, Wright SW, Bolch CJS, Nichols PD, Skerratt JH, McMinn A. Antarctic Distribution, Pigment and Lipid Composition, and Molecular Identification of the Brine Dinoflagellate *Polarella* *Glacialis* (dinophyceae)1. J Phycol. 2004;40: 867–873. doi:[10.1111/j.1529-8817.2004.03169.x](https://doi.org/10.1111/j.1529-8817.2004.03169.x)

137. Wang J, Zhu J, Liu S, Liu B, Gao Y, Wu Z. Generation of reactive oxygen species in cyanobacteria and green algae induced by allelochemicals of submerged macrophytes. Chemosphere. 2011;85: 977–982. doi:[10.1016/j.chemosphere.2011.06.076](https://doi.org/10.1016/j.chemosphere.2011.06.076)

138. Zingone A, Forlani G, Percopo I, Montresor M. Morphological characterization of *Phaeocystis* *Antarctica* (Prymnesiophyceae). Phycologia. 2011;50: 650–660. doi:[10.2216/11-36.1](https://doi.org/10.2216/11-36.1)

139. Shields AR, Smith WO. Size-fractionated photosynthesis/irradiance relationships during Phaeocystis antarctica-dominated blooms in the Ross Sea, Antarctica. J Plankton Res. 2009;31: 701–712. doi:[10.1093/plankt/fbp022](https://doi.org/10.1093/plankt/fbp022)

140. Peperzak L, Colijn F, Vrieling EG, Gieskes WWC, Peeters JCH. Observations of flagellates in colonies of *Phaeocystis* *Globosa* (Prymnesiophyceae); a hypothesis for their position in the life cycle. J Plankton Res. 2000;22: 2181–2203. doi:[10.1093/plankt/22.12.2181](https://doi.org/10.1093/plankt/22.12.2181)

141. Smayda TJ. Turbulence, watermass stratification and harmful algal blooms: An alternative view and frontal zones as “pelagic seed banks.” Harmful Algae. 2002;1: 95–112. doi:[10.1016/S1568-9883(02)00010-0](https://doi.org/10.1016/S1568-9883(02)00010-0)

142. Garduño RA, Hall BD, Brown L, Robinson MG. Two Distinct Colonial Morphotypes of *Amphora* *Coffeaeformis* (bacillariophyceae) Cultured on Solid Media. J Phycol. 1996;32: 469–478. doi:[10.1111/j.0022-3646.1996.00469.x](https://doi.org/10.1111/j.0022-3646.1996.00469.x)

143. Kaczmarska I, Mather L, Luddington IA, Muise F, Ehrman JM. Cryptic diversity in a cosmopolitan diatom known as *Asterionellopsis* *Glacialis* (Fragilariaceae): Implications for ecology, biogeography, and taxonomy. Am J Bot. 2014;101: 267–286. doi:[10.3732/ajb.1300306](https://doi.org/10.3732/ajb.1300306)

144. Dąbek P, Ashworth MP, Górecka E, Krzywda M, Bornman TG, Sato S, et al. Toward a multigene phylogeny of the Cymatosiraceae (Bacillariophyta, Mediophyceae) II: Morphological and molecular insights into the taxonomy of the forgotten species *Campylosira* *Africana* and of *Extubocellulus* , with a description of two new taxa. J Phycol. 2019;55: 425–441. doi:[10.1111/jpy.12831](https://doi.org/10.1111/jpy.12831)

145. Rae BD, Long BM, Whitehead LF, Förster B, Badger MR, Price GD. Cyanobacterial Carboxysomes: Microcompartments that Facilitate CO _2_ Fixation. J Mol Microbiol Biotechnol. 2013;23: 300–307. doi:[10.1159/000351342](https://doi.org/10.1159/000351342)

146. Chung I-K, Kang Y-H. A Marine Picophytoplankter from Korea: *Pycnococcus* *Provasolii* Guillard. Journal of the korean society of oceanography. 1996;31: 150–154.

147. Kvernvik AC, Rokitta SD, Leu E, Harms L, Gabrielsen TM, Rost B, et al. Higher sensitivity towards light stress and ocean acidification in an Arctic sea-ice-associated diatom compared to a pelagic diatom. New Phytologist. 2020;226: 1708–1724. doi:[10.1111/nph.16501](https://doi.org/10.1111/nph.16501)

148. Olenina I, Hajdu S, Edler L, Andersson A, Wasmund N, Busch S, et al. Biovolumes and Size-Casses of Phytoplankton in the Baltic Sea. Baltic Marine Environment Protection Commission; 2006. Report No.: 106.

149. Rokitta S. Transcriptome assemblies of *Thalassiosira* *Hyalina* and *Nitzschia* *Frigida*. Zenodo; 2019. doi:[10.5281/zenodo.3361258](https://doi.org/10.5281/zenodo.3361258)

150. Rozanska M, Gosselin M, Poulin M, Wiktor J, Michel C. Influence of environmental factors on the development of bottom ice protist communities during the winterspring transition. Mar Ecol Prog Ser. 2009;386: 43–59. doi:[10.3354/meps08092](https://doi.org/10.3354/meps08092)

151. Hegseth EN, Sundfjord A. Intrusion and blooming of Atlantic phytoplankton species in the high Arctic. J Mar Syst. 2008;74: 108–119. doi:[10.1016/j.jmarsys.2007.11.011](https://doi.org/10.1016/j.jmarsys.2007.11.011)

152. Webb EA, Ehrenreich IM, Brown SL, Valois FW, Waterbury JB. Phenotypic and genotypic characterization of multiple strains of the diazotrophic cyanobacterium, *Crocosphaera* *Watsonii*, isolated from the open ocean. Environmental Microbiology. 2009;11: 338–348. doi:[10.1111/j.1462-2920.2008.01771.x](https://doi.org/10.1111/j.1462-2920.2008.01771.x)

153. Bench S, Ilikchyan I, Tripp H, Zehr J. Two Strains of *Crocosphaera* *Watsonii* with Highly Conserved Genomes are Distinguished by Strain-Specific Features. Front Microbiol. 2011;2: 261. doi:[10.3389/fmicb.2011.00261](https://doi.org/10.3389/fmicb.2011.00261)

154. Foster RA, Sztejrenszus S, Kuypers MMM. Measuring carbon and N2 fixation in field populations of colonial and free-living unicellular cyanobacteria using nanometer-scale secondary ion mass Spectrometry1. J Phycol. 2013;49: 502–516. doi:[10.1111/jpy.12057](https://doi.org/10.1111/jpy.12057)

155. Beer S, Björk M, Beardall J. Photosynthesis in the marine environment. Second edition. Ames, Iowa : Chichester, West Sussex, UK: John Wiley & Sons, Inc; 2014.

156. Welsh EA, Liberton M, Stöckel J, Loh T, Elvitigala T, Wang C, et al. The genome of *Cyanothece* 51142, a unicellular diazotrophic cyanobacterium important in the marine nitrogen cycle. Proc Natl Acad Sci U S A. 2008;105: 15094–15099. doi:[10.1073/pnas.0805418105](https://doi.org/10.1073/pnas.0805418105)

157. Mareš J, Johansen JR, Hauer T, Zima Jr. J, Ventura S, Cuzman O, et al. Taxonomic resolution of the genus *Cyanothece* (Chroococcales, Cyanobacteria), with a treatment on *Gloeothece* and three new genera, *Crocosphaera*, *Rippkaea*, and *Zehria*. J Phycol. 2019;55: 578–610. doi:[10.1111/jpy.12853](https://doi.org/10.1111/jpy.12853)

158. Kloster M, Kauer G, Esper O, Fuchs N, Beszteri B. Morphometry of the diatom *Fragilariopsis* *Kerguelensis* from Southern Ocean sediment: High-throughput measurements show second morphotype occurring during glacials. Marine Micropaleontology. 2018;143: 70–79. doi:[10.1016/j.marmicro.2018.07.002](https://doi.org/10.1016/j.marmicro.2018.07.002)

159. Cortese G, Gersonde R. Morphometric variability in the diatom Fragilariopsis kerguelensis: Implications for Southern Ocean paleoceanography. Earth and Planetary Science Letters. 2007;257: 526–544. doi:[10.1016/j.epsl.2007.03.021](https://doi.org/10.1016/j.epsl.2007.03.021)

160. Cefarelli AO, Ferrario ME, Almandoz GO, Atencio AG, Akselman R, Vernet M. Diversity of the diatom genus *Fragilariopsis* in the Argentine Sea and Antarctic waters: Morphology, distribution and abundance. Polar Biol. 2010;33: 1463–1484. doi:[10.1007/s00300-010-0794-z](https://doi.org/10.1007/s00300-010-0794-z)

161. Cusack C, Raine R, Patching JW. Occurrence of Species from the Genus *Pseudo-nitzschia* Peragallo in Irish Waters. Biology and Environment: Proceedings of the Royal Irish Academy. 2004;104B: 55–74. Available: <https://www.jstor.org/stable/20500205>

162. Rines J, Donaghay P, Dekshenieks M, Sullivan J, Twardowski M. Thin layers and camouflage: Hidden *Pseudo-nitzschia* *Spp.* (Bacillariophyceae) populations in a fjord in the San Juan Islands, Washington, USA. Mar Ecol Prog Ser. 2002;225: 123–137. doi:[10.3354/meps225123](https://doi.org/10.3354/meps225123)

163. Hernández-Becerril DU. Species of the planktonic diatom genus *Pseudo-nitzschia* of the Pacific coasts of Mexico. Hydrobiologia. 1998;379: 77–84. doi:[10.1023/A:1003471828302](https://doi.org/10.1023/A:1003471828302)

164. Shetye SS, Mohan R, Patil S, Kumar A. Diatom distribution in the Enderby Basin, East Antarctica. Polar Science. 2021; 100748. doi:[10.1016/j.polar.2021.100748](https://doi.org/10.1016/j.polar.2021.100748)

165. Hasle GR, Semina HJ. The Marine Planktonic Diatoms *Thalassiothrix* *Longissima*$<\setminus$*i*$>$ *and* *Thalassiothrix* *Antarctica* *with Comments onThalassionema* *Spp.* *and* *Synedra* *Reinboldii*. Diatom Research. 1987;2: 175–192. doi:[10.1080/0269249X.1987.9704996](https://doi.org/10.1080/0269249X.1987.9704996)

166. Hasle GR. The Marine, Planktonic Diatom Family Thalassionemataceae: Morphology, Taxonomy and Distribution. Diatom Research. 2001;16: 1–82. doi:[10.1080/0269249X.2001.9705509](https://doi.org/10.1080/0269249X.2001.9705509)

167. Johansson ON, Pinder MIM, Ohlsson F, Egardt J, Töpel M, Clarke AK. Friends With Benefits: Exploring the Phycosphere of the Marine Diatom *Skeletonema* *Marinoi*. Front Microbiol. 2019;10: 1828. doi:[10.3389/fmicb.2019.01828](https://doi.org/10.3389/fmicb.2019.01828)

168. Bo W, Baihui C, Qi L, Quanxi W. Morphological description of the Genus *Skeletonema* *(Bacillariophyceae)* in Yangtze River Estuary，China. Journal of Shanghai Normal University( Natural Sciences). 2013;42: 6.

169. Amato A, Sabatino V, Nylund GM, Bergkvist J, Basu S, Andersson MX, et al. Grazer-induced transcriptomic and metabolomic response of the chain-forming diatom *Skeletonema* *Marinoi*. ISME J. 2018;12: 1594–1604. doi:[10.1038/s41396-018-0094-0](https://doi.org/10.1038/s41396-018-0094-0)

170. Jung S-W, Yun S-M, Lee S-D, Kim Y-O, Lee J-H. Morphological Characteristics of Four Species in the Genus *Skeletonema* in Coastal Waters of South Korea. ALGAE. 2009;24: 195–203. doi:[10.4490/ALGAE.2009.24.4.195](https://doi.org/10.4490/ALGAE.2009.24.4.195)

171. Cheng J, Li Y, Liang J, Gao Y, Wang P, Kin-Chung H, et al. Morphological variability and genetic diversity in five species of Skeletonema (Bacillariophyta). Progress in Natural Science. 2008;18: 1345–1355. doi:[10.1016/j.pnsc.2008.05.002](https://doi.org/10.1016/j.pnsc.2008.05.002)

172. Biquand E, Okubo N, Aihara Y, Rolland V, Hayward DC, Hatta M, et al. Acceptable symbiont cell size differs among cnidarian species and may limit symbiont diversity. The ISME Journal. 2017;11: 1702–1712. doi:[10.1038/ismej.2017.17](https://doi.org/10.1038/ismej.2017.17)

173. Aranda M, Li Y, Liew YJ, Baumgarten S, Simakov O, Wilson MC, et al. Genomes of coral dinoflagellate symbionts highlight evolutionary adaptations conducive to a symbiotic lifestyle. Scientific Reports. 2016;6. doi:[10.1038/srep39734](https://doi.org/10.1038/srep39734)

174. Lin S, Cheng S, Song B, Zhong X, Lin X, Li W, et al. The *Symbiodinium* *Kawagutii* genome illuminates dinoflagellate gene expression and coral symbiosis. Science. 2015;350: 691–694. doi:[10.1126/science.aad0408](https://doi.org/10.1126/science.aad0408)

175. Lee SY, Jeong HJ, Kang NS, Jang TY, Jang SH, Lajeunesse TC. *Symbiodinium Tridacnidorum* Sp. Nov., A Dinoflagellate Common to Indo-Pacific Giant Clams, and a Revised Morphological Description of *Symbiodinium* *Microadriaticum* Freudenthal, Emended Trench & Blank. European Journal of Phycology. 2015;50: 155–172. doi:[10.1080/09670262.2015.1018336](https://doi.org/10.1080/09670262.2015.1018336)

176. van Baren MJ, Bachy C, Reistetter EN, Purvine SO, Grimwood J, Sudek S, et al. Evidence-based green algal genomics reveals marine diversity and ancestral characteristics of land plants. BMC Genomics. 2016;17. doi:[10.1186/s12864-016-2585-6](https://doi.org/10.1186/s12864-016-2585-6)

177. Simon N, Foulon E, Grulois D, Six C, Desdevises Y, Latimier M, et al. Revision of the Genus Micromonas Manton et Parke (Chlorophyta, Mamiellophyceae), of the Type Species *M. Pusilla* (Butcher) Manton & Parke and of the Species *M. Commoda* van Baren, Bachy and Worden and Description of Two New Species Based on the Genetic and Phenotypic Characterization of Cultured Isolates. Protist. 2017;168: 612–635. doi:[10.1016/j.protis.2017.09.002](https://doi.org/10.1016/j.protis.2017.09.002)

178. Lovejoy C. Small Planktonic single celled eukaryotes from the Arctic Ocean. 2010. doi:[10.25585/1488049](https://doi.org/10.25585/1488049)

179. Figueroa R, Garcés E, Massana R, Camp J. Description, Host-specificity, and Strain Selectivity of the Dinoflagellate Parasite *Parvilucifera* *Sinerae* sp. Nov. (Perkinsozoa). Protist. 2008;159: 563–578. doi:[10.1016/j.protis.2008.05.003](https://doi.org/10.1016/j.protis.2008.05.003)

180. Thomsen HA. An ultrastructural survey of the chrysophycean genus *Paraphysomonas* under natural conditions. British Phycological Journal. 1975;10: 113–127. doi:[10.1080/00071617500650111](https://doi.org/10.1080/00071617500650111)

181. Demura M, Noël M-H, Kasai F, Watanabe MM, Kawachi M. Taxonomic revision of *Chattonella* *Antiqua*, *C. Marina* and *C. Ovata* (Raphidophyceae) based on their morphological characteristics and genetic diversity. Phycologia. 2009;48: 518–535. doi:[10.2216/08-98.1](https://doi.org/10.2216/08-98.1)

182. Shikata T, Takahashi F, Nishide H, Shigenobu S, Kamei Y, Sakamoto S, et al. RNA-Seq Analysis Reveals Genes Related to Photoreception, Nutrient Uptake, and Toxicity in a Noxious Red-Tide Raphidophyte *Chattonella* *Antiqua*. Front Microbiol. 2019;10. doi:[10.3389/fmicb.2019.01764](https://doi.org/10.3389/fmicb.2019.01764)

183. Horiguchi T. *Heterocapsa Circularisquama* sp. Nov. (Peridiniales, Dinophyceae): A new marine dinoflagellate causing mass mortality of bivalves in Japan. Phycological Research. 1995;43: 129–136. doi:[10.1111/j.1440-1835.1995.tb00016.x](https://doi.org/10.1111/j.1440-1835.1995.tb00016.x)

184. Chepurnov VA, Mann DG, Dassow P von, Vanormelingen P, Gillard J, Inzé D, et al. In search of new tractable diatoms for experimental biology. BioEssays. 2008;30: 692–702. doi:[10.1002/bies.20773](https://doi.org/10.1002/bies.20773)

185. Osuna-Cruz CM, Bilcke G, Vancaester E, De Decker S, Bones AM, Winge P, et al. The *Seminavis* *Robusta* genome provides insights into the evolutionary adaptations of benthic diatoms. Nat Commun. 2020;11: 3320. doi:[10.1038/s41467-020-17191-8](https://doi.org/10.1038/s41467-020-17191-8)

186. Figueroa RI, Bravo I, Fraga S, Garcés E, Llaveria G. The Life History and Cell Cycle of *Kryptoperidinium* *Foliaceum*, A Dinoflagellate with Two Eukaryotic Nuclei. Protist. 2009;160: 285–300. doi:[10.1016/j.protis.2008.12.003](https://doi.org/10.1016/j.protis.2008.12.003)

187. Moldrup M, Moestrup Ø, Hansen PJ. Loss of Phototaxis and Degeneration of an Eyespot in Long-term Algal Cultures: Evidence from Ultrastructure and Behaviour in the Dinoflagellate *Kryptoperidinium* *Foliaceum*. Journal of Eukaryotic Microbiology. 2013;60: 327–334. doi:[10.1111/jeu.12036](https://doi.org/10.1111/jeu.12036)

188. Foflonker F, Price DC, Qiu H, Palenik B, Wang S, Bhattacharya D. Genome of the halotolerant green alga *Picochlorum* *Sp.* Reveals strategies for thriving under fluctuating environmental conditions. Environmental Microbiology. 2015;17: 412–426. doi:[10.1111/1462-2920.12541](https://doi.org/10.1111/1462-2920.12541)

189. Foflonker F, Mollegard D, Ong M, Yoon HS, Bhattacharya D. Genomic Analysis of Picochlorum Species Reveals How Microalgae May Adapt to Variable Environments. Molecular Biology and Evolution. 2018;35: 2702–2711. doi:[10.1093/molbev/msy167](https://doi.org/10.1093/molbev/msy167)

190. Oliveira M. Magnetic Stimulation on the Growth of the Microalga *Nannochloropsis* *Oculata*. Master of {{Engineering Science}} Degree in {{Chemical}} and {{Biochemical Engineering}}, The University of Western Ontario. 2017.

191. Hulatt CJ, Wijffels RH, Posewitz MC. The Genome of the Haptophyte *Diacronema* *Lutheri* (*Pavlova* *Lutheri*, Pavlovales): A Model for Lipid Biosynthesis in Eukaryotic Algae. Genome Biology and Evolution. 2021;13: evab178. doi:[10.1093/gbe/evab178](https://doi.org/10.1093/gbe/evab178)

192. Hongo Y, Kimura K, Takaki Y, Yoshida Y, Baba S, Kobayashi G, et al. The genome of the diatom *Chaetoceros* *Tenuissimus* carries an ancient integrated fragment of an extant virus. Sci Rep. 2021;11: 22877. doi:[10.1038/s41598-021-00565-3](https://doi.org/10.1038/s41598-021-00565-3)

193. Füssy Z, Masařová P, Kručinská J, Esson HJ, Oborník M. Budding of the Alveolate Alga *Vitrella* *Brassicaformis* Resembles Sexual and Asexual Processes in Apicomplexan Parasites. Protist. 2017;168: 80–91. doi:[10.1016/j.protis.2016.12.001](https://doi.org/10.1016/j.protis.2016.12.001)

194. Woo YH, Ansari H, Otto TD, Klinger CM, Kolisko M, Michálek J, et al. Chromerid genomes reveal the evolutionary path from photosynthetic algae to obligate intracellular parasites. eLife. 2015;4: e06974. doi:[10.7554/eLife.06974](https://doi.org/10.7554/eLife.06974)

195. Oborník M, Modrý D, Lukeš M, Černotíková-Stříbrná E, Cihlář J, Tesařová M, et al. Morphology, Ultrastructure and Life Cycle of *Vitrella* *Brassicaformis* n. Sp., N. Gen., A Novel Chromerid from the Great Barrier Reef. Protist. 2012;163: 306–323. doi:[10.1016/j.protis.2011.09.001](https://doi.org/10.1016/j.protis.2011.09.001)

196. Lemieux C, Turmel M, Otis C, Pombert J-F. A streamlined and predominantly diploid genome in the tiny marine green alga *Chloropicon* *Primus*. Nat Commun. 2019;10: 4061. doi:[10.1038/s41467-019-12014-x](https://doi.org/10.1038/s41467-019-12014-x)
